# Restoring Atrial T-Tubules Augments Systolic Ca Upon Recovery From Heart Failure

**DOI:** 10.1161/CIRCRESAHA.124.324601

**Published:** 2024-08-14

**Authors:** Jessica L. Caldwell, Jessica D. Clarke, Charlotte E.R. Smith, Christian Pinali, Callum J. Quinn, Charles M. Pearman, Aiste Adomaviciene, Emma J. Radcliffe, Amy Watkins, Margaux A Horn, Elizabeth F. Bode, George W.P. Madders, Mark Eisner, David A. Eisner, Andrew W. Trafford, Katharine M. Dibb

**Affiliations:** Unit of Cardiac Physiology, Manchester Academic Health Science Centre, University of Manchester, United Kingdom.

**Keywords:** calcium, heart failure, heart diseases, myocytes, cardiac, sarcoplasmic reticulum, volume electron microscopy

## Abstract

**BACKGROUND:**

Transverse (t)-tubules drive the rapid and synchronous Ca^2+^ rise in cardiac myocytes. The virtual complete atrial t-tubule loss in heart failure (HF) decreases Ca^2+^ release. It is unknown if or how atrial t-tubules can be restored and how this affects systolic Ca^2+^.

**METHODS:**

HF was induced in sheep by rapid ventricular pacing and recovered following termination of rapid pacing. Serial block-face scanning electron microscopy and confocal imaging were used to study t-tubule ultrastructure. Function was assessed using patch clamp, Ca^2+^, and confocal imaging. Candidate proteins involved in atrial t-tubule recovery were identified by western blot and expressed in rat neonatal ventricular myocytes to determine if they altered t-tubule structure.

**RESULTS:**

Atrial t-tubules were lost in HF but reappeared following recovery from HF. Recovered t-tubules were disordered, adopting distinct morphologies with increased t-tubule length and branching. T-tubule disorder was associated with mitochondrial disorder. Recovered t-tubules were functional, triggering Ca^2+^ release in the cell interior. Systolic Ca^2+^, *I*_Ca-L_, sarcoplasmic reticulum Ca^2+^ content, and sarcoendoplasmic reticulum Ca^2+^ ATPase function were restored following recovery from HF. Confocal microscopy showed fragmentation of ryanodine receptor staining and movement away from the z-line in HF, which was reversed following recovery from HF. Acute detubulation, to remove recovered t-tubules, confirmed their key role in restoration of the systolic Ca^2+^ transient, the rate of Ca^2+^ removal, and the peak L-type Ca^2+^ current. The abundance of telethonin and myotubularin decreased during HF and increased during recovery. Transfection with these proteins altered the density and structure of tubules in neonatal myocytes. Myotubularin had a greater effect, increasing tubule length and branching, replicating that seen in the recovery atria.

**CONCLUSIONS:**

We show that recovery from HF restores atrial t-tubules, and this promotes recovery of *I*_Ca-L_, sarcoplasmic reticulum Ca^2+^ content, and systolic Ca^2+^. We demonstrate an important role for myotubularin in t-tubule restoration. Our findings reveal a new and viable therapeutic strategy.

**Novelty and Significance**

**What Is Known?**

- Contraction of cardiac cells is brought about by a rapid rise in intracellular calcium, called the calcium transient, which is driven by a coordinated release of calcium from intracellular stores.
- Transverse-tubules (t-tubules) are deep invaginations of the surface membrane of atrial and ventricular cells bring which calcium channels, the trigger for calcium release, into close apposition with intracellular calcium stores, facilitating the calcium transient.
- In heart failure, virtually all atrial t-tubules are lost, contributing to the decreased calcium transient and contraction, but it is unknown if atrial t-tubules and the calcium transient can be restored.

**What New Information Does This Article Contribute?**

- Atrial t-tubules can be restored following their loss in heart failure.
- Newly formed t-tubules have a disorganized structure but are functional and underlie the restoration of key calcium handling processes and the recovery of the calcium transient.
- The lipid phosphatase myotubularin drives the growth of longer, more branched t-tubules and likely plays a key role in t-tubule restoration following recovery from heart failure, revealing a viable therapeutic target.

T-tubule disruption in heart disease decreases calcium release in the cell interior, thus playing an important role in the decreased calcium transient amplitude and contractile dysfunction. Interventions such as exercise or resynchronization therapy have been shown to partially restore ventricular t-tubules and the calcium transient. However, since new ventricular t-tubules cannot be distinguished from preexisting t-tubules, whether generating new t-tubules can restore calcium handling is unknown. The almost complete loss of atrial t-tubules in our model of tachypacing induced heart failure allows us to study t-tubule recovery upon termination of tachypacing and address if new t-tubules can form and if this is therapeutically desirable. We show newly formed atrial t-tubules can restore t-tubule density, but new t-tubules are structurally disordered, being longer and more branched. Importantly, new atrial t-tubules restore the atrial calcium transient. Our data suggest the t-tubule–associated protein myotubularin drives the recovery of longer, more branched t-tubules. The demonstration that new atrial t-tubules can fully restore the calcium transient is significant, highlighting a potential future therapeutic pathway utilizing the lipid phosphatase myotubularin.

**In This Issue, see p 705**

**Meet the First Author, see p 706**

**Editorial, see p 755**

Transverse-tubules (t-tubules) are invaginations of the surface membrane of cardiac myocytes that synchronize the systolic rise of [Ca^2+^]_i_.^1^ Ventricular t-tubule loss occurs in various cardiovascular diseases, reducing the amplitude and synchronicity of the systolic Ca^2+^ transient.^1,2^ The extent of t-tubule loss correlates with left ventricular dysfunction.^3,4^ Large mammals, including humans, have extensive atrial t-tubule networks, which are reduced in heart failure (HF) and atrial fibrillation.^5–8^ However, in contrast to the ≈24% reduction in ventricular t-tubules,^2^ HF causes near-complete loss of atrial t-tubules with profound effects on systolic Ca^2+^.^5,8^

T-tubules can be at least partially restored in the failing left ventricle by mechanical unloading,^9^ *SERCA2a* gene therapy,^10^ resynchronization therapy,^11^ treadmill exercise,^12^ β-blockers,^13^ and phosphodiesterase-5 inhibition.^14^ Some studies have also shown that this is accompanied by restoration of systolic Ca^2+^.^9–11,14,15^ However, since only a minority of ventricular t-tubules are lost,^1,8,10^ newly formed t-tubules cannot be distinguished from preexisting t-tubules. Therefore, whether new t-tubules are functional and whether the restoration of systolic Ca^2+^ is due to new t-tubules rather than enhanced Ca^2+^ release of preexisting t-tubules remains unknown. This information is essential to understand whether t-tubule restoration has the potential to restore Ca^2+^ handling and contractile function in disease. Uniquely, the near-complete loss of atrial t-tubules in HF^5,8^ allows us to determine if t-tubule restoration is possible and if new atrial t-tubules can trigger Ca^2+^ release and restore Ca^2+^ handling necessary for restoration of systolic Ca^2+^.

The process by which t-tubules are formed is not well understood. Many proteins have been implicated in the ventricle, including JPH2 (junctophilin 2),^16^ Amp-II (amphiphysin II, BIN1 [bridging integrator 1]),^8,17^ and Tcap (telethonin),^18^ but their role in the atria is unknown. Therefore, the main aims of this study were to establish in the atrium: (1) if new t-tubules can form once they have been lost in HF; (2) if new t-tubules can restore *I*_Ca-L_ and trigger Ca^2+^ release to recover the systolic Ca^2+^ transient; and (3) which proteins drive the formation of new t-tubules.

## METHODS

### Data Availability

The data that supports the findings of this study are available from the corresponding author upon reasonable request.

For detailed methods and the major resources table, please see Supplemental Material.

The study accords with the United Kingdom Animals (Scientific Procedures) Act, 1986, European Union Directive (EU/2010/63) and is reported in accordance with the ARRIVE guidelines. Institutional approval was obtained from the University of Manchester Animal Welfare and Ethical Review Board.

## RESULTS

### Atrial T-Tubules Are Restored Following Recovery From HF

**Figure 1.**
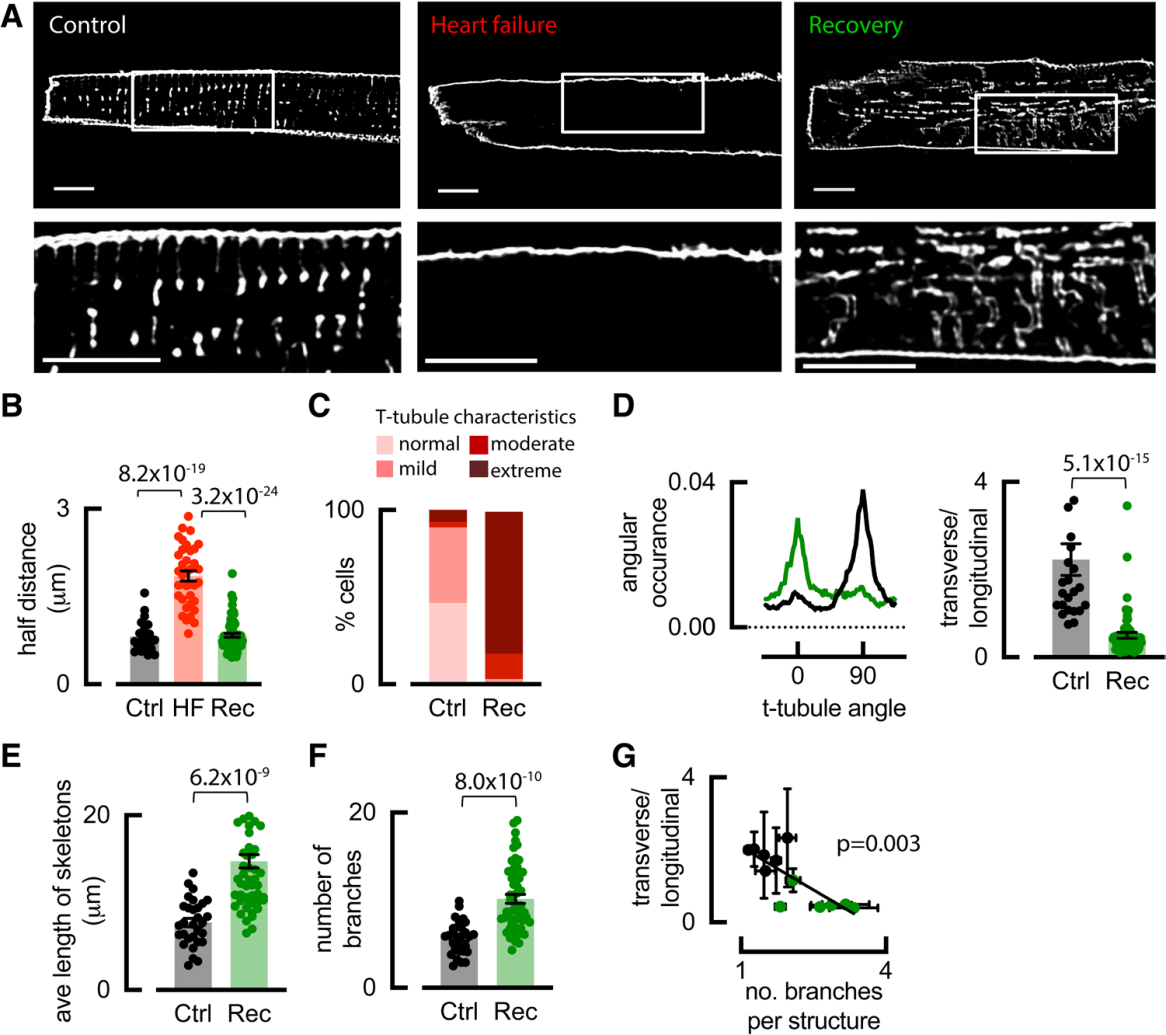
**Sheep atrial t-tubules are restored following recovery (Rec) from heart failure (HF). A**, Di-4-ANEPPS staining of sheep atrial myocytes showing t-tubule loss in HF and restoration in recovery. **B**, Half distance to the nearest t-tubule and surface membrane. **C**, Categorization of t-tubule disorder. **D**, Typical control (black) and recovery (green) t-tubule orientation; transverse tubules (90) and longitudinal (0) and transverse:longitudinal tubule ratio. Mean data were calculated from skeletonized images for (**E**) t-tubule length and (**F**) branching. **G**, Relationship between branching and t-tubule angle (R^2^=0.614). Symbols denote cells n; control: n=24 (6 animals) for **B** to **D**, and **G**, n=30 (7 animals) for **E** and **F**; HF: n=36 (3 animals); recovery: n=54 (6 animals) for **E** to **G**, n=61 (6 animals) for **B** to **D**; compared using linear mixed modeling for **B**, and **D** to **F** or simple linear regression for **G**. Data are presented as mean±SEM. Scale bars: 10 µm. Ctrl indicates control.

As shown previously, HF produced by ventricular tachypacing is associated with ventricular dilatation and reduced contractility^5,8,14,19^ (Table S1). Figure 1A shows that in the atria, t-tubules are present in control but almost completely lost in HF.^5^ Sheep developed clinical signs of HF, including lethargy, dyspnea, and weight loss, (after 39±3.3 days; range, 16–85 days). Rapid ventricular pacing was then discontinued, resulting in reversal of subjective signs of HF and partial restoration of ventricular contractility within 37±1.2 days (Table S1). This recovery coincided with the reversal of atrial cellular hypertrophy (Table S1) and the reappearance of atrial t-tubules (Figure 1A, right panel). T-tubule density was assessed from the distance of points in the cell to the nearest membrane in the x, y, or z direction.^5^ This distance increased in HF when t-tubules were lost but was restored to control levels on recovery, demonstrating atrial t-tubule restoration post-HF is achievable (Figure 1B). This model therefore provides an opportunity to understand the mechanisms and functional consequences of restoring atrial t-tubules.

### Recovered Atrial T-Tubules Are Disordered

Despite restoration of atrial t-tubule density, t-tubule organization was dramatically altered in atrial cells following recovery from HF (henceforth termed recovery cells; Figure 1A). First, as described in Supplemental Methods, we classified t-tubule disorder severity into 4 groups: normal, mild, moderate, and extreme disorganization. Extreme t-tubule disorganization increased from only 2% in control to 79% following recovery (blinded observations; Figure 1C). To characterize the underlying structural changes, t-tubule images were skeletonized,^8^ revealing a shift from mostly transversely oriented (90°) in control to predominantly longitudinal (0°) t-tubules in recovery cells (Figure 1D), contributing to the t-tubule disorder. Importantly, t-tubules in recovery cells were longer (Figure 1E) and more branched (Figure 1F), further increasing disorder. Animals with greater t-tubule branching also contained the highest proportion of longitudinally oriented tubules (Figure 1G).

**Figure 2.**
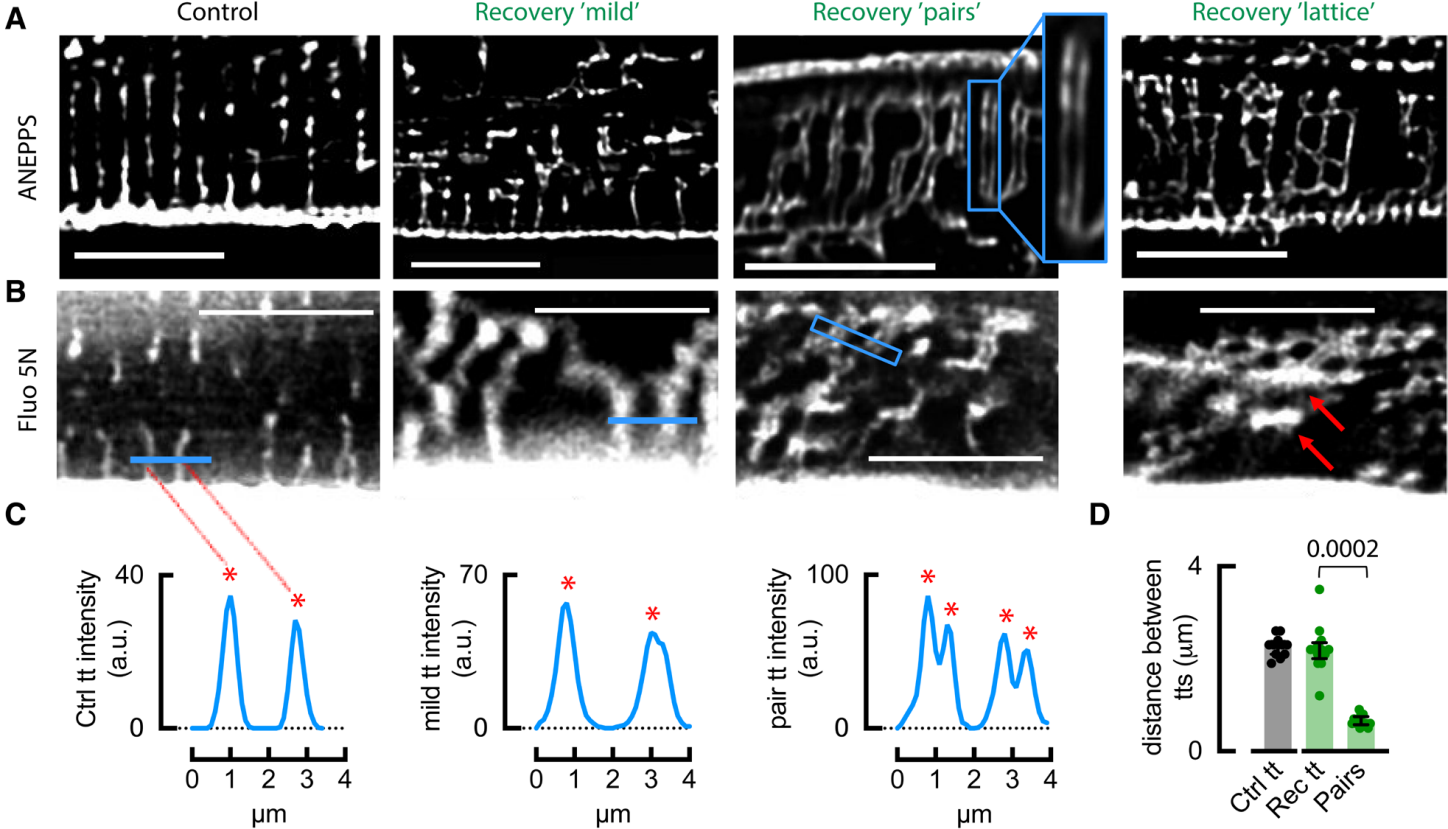
**Recovered atrial t-tubules are disordered. A**, Di-4-ANEPPS staining of control and recovered atrial myocytes. **B**, Application of fluo-5N reveals t-tubules open to the cell exterior, occurring in pairs (blue box). **C**, Tubule pairs were confirmed through intensity plots corresponding to blue lines/boxes in **B**. Red asterisks denote peaks corresponding to t-tubules. **D**, Spacing of t-tubules and individual t-tubules forming pairs (red arrows). Symbols denote cells: n=12 (1 animal) for control, n=13 (2 animals) for recovery (3 tubules averaged per cell); compared using Wilcoxon signed-rank test with multiple comparisons. Data are presented as median±IQR. Scale bars: 10 µm. Ctrl indicates control; IQR, interquartile range; Rec, recovery; and tt, t-tubule.

Figure 2A illustrates the altered appearance of recovered atrial t-tubules, displaying visible t-tubule pairs and lattice-like structures. In some animals, the disorder was mild (Figure 2A, second panel), and while there are many transverse structures, there were also several longitudinal ones. One concern with using aminonaphthylethenylpyridinium (ANEP) staining is that it does not prove that these structures are tubules with a patent lumen. To confirm the nature of these structures, we therefore added the cell-impermeant fluorescent Ca^2+^ indicator fluo-5N to the extracellular solution (Figure 2B). Fluo-5N entered both control and recovered t-tubules (Figure 2B), indicating their continuity with the cell exterior and potential functional importance. Notably, closely associated pairs of tubules were observed (Figure 2A, third panel) and the fluo-5N did not fill the space between parallel transverse tubule pairs (blue box in Figure 2B, third panel), suggesting separate tubules in close proximity as opposed to a poorly resolved larger structure. Normal t-tubules were on average 2.27±0.1 µm apart consistent with a z-line localization (Figure 2C and 2D).^20^ When occurring in pairs, individual t-tubules were only 0.65±0.03 µm apart (Figure 2C and 2D), indicating non-z-line localization. Confocal microscopy also revealed lattices of tubules (Figure 2A, right panel), which often contained dilated, longitudinally oriented tubules (red arrows in Figure 2B, right panel), which filled with fluo-5N, indicating continuity with the surface membrane and potential functionality. Therefore, recovered t-tubules often exhibited high disorder, longitudinal orientation, variable width, paired, and lattice-like structures. Confocal microscopy lacks the resolution to understand the 3-dimensional structure of these recovered t-tubules, so subsequent experiments used serial block-face scanning electron microscopy (sbfSEM).

### Recovered Atrial T-Tubules Are Large and Exhibit Extreme Structural Disorder Associated With Disrupted Mitochondrial Positioning

**Figure 3.**
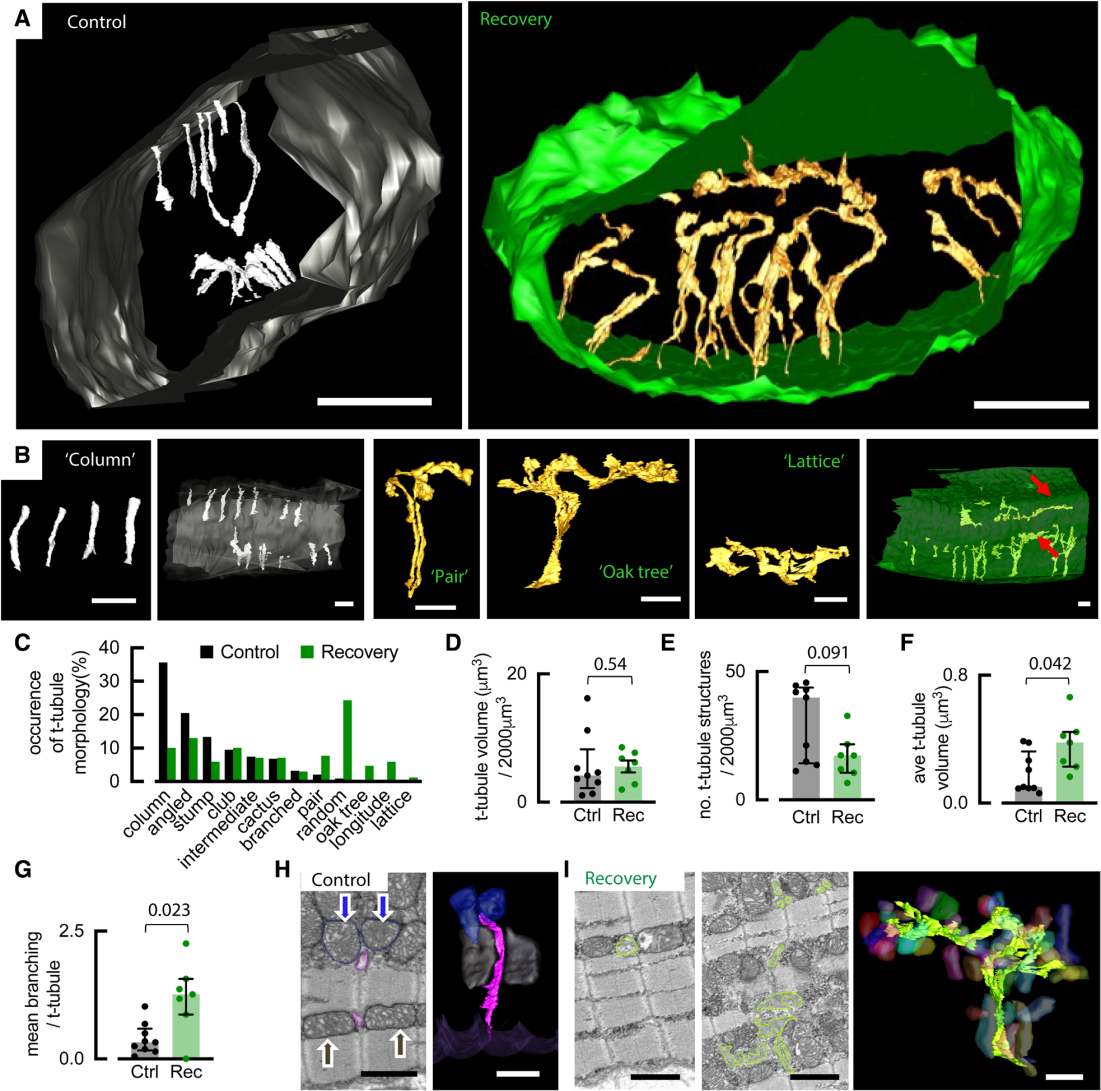
**The diversity of atrial t-tubule remodeling is associated with mitochondrial positioning. A**, sbfSEM 3-dimensional reconstructions of control (Ctrl) and recovery (Rec) atrial myocytes (scale bars: 5 µm). **B**, Common t-tubule morphologies and longitudinal cell views for Ctrl (white) and Rec (gold) with red arrows indicating oak tree and lattice morphologies (scale bars: 2 µm). **C**, Percentage occurrence of the most common t-tubule morphologies. Mean data from serial block-face scanning electron microscopy (sbfSEM) for (**D**) t-tubule volume, (**E**) number of t-tubules per volume, (**F**) average t-tubule volume, and (**G**) average branching per t-tubule. Symbols denote cells; n=9 for Ctrl and n=7 for Rec (3 animals for each group) for **D** to **G**; compared using Mann-Whitney *U* test with multiple comparisons. Data are presented as median±IQR. Example 2-dimensional sections and models of (**H**) control atrial t-tubule (pink) extending along the z-line between a single band of mitochondria (gray arrows/mitochondria) but not penetrating the thicker mitochondrial bed (blue arrows/mitochondria) and (**I**) a recovered atrial t-tubule (lime), which penetrates thick mitochondrial beds forming irregular branching (scale bars: 2 µm); n=9 cells from 3 animals for Ctrl and Rec.

Using enhanced sbfSEM resolution, we first explored the ultrastructure of control atrial t-tubules, uncovering novel characteristics. We found sheep atrial t-tubules occupy ≈0.27% cell volume, project radially from the sarcolemma primarily at z-lines (as in human atrial myocytes^6^), and display morphological heterogeneity (Figure 3A, left; Table S2). In contrast to relatively uniform ventricular t-tubules,^21,22^ control atrial t-tubules exhibit unique morphologies, including stump and club shapes (Figure 3A; Table S2; Figure S1A and S1B).

Consistent with confocal data, recovered atrial t-tubules were highly disordered (Figure 3A, right). Normal t-tubule morphologies in control cells (white) were replaced by disordered structures in recovery cells (gold), for example, random, oak tree, longitudinal, lattice, and t-tubule pairs (Figure 3B), which together accounted for 61% of the total t-tubule volume in recovery (Figure 3C; Figure S1A and S1B). Recovered tubules, such as the oak tree and lattice morphologies, often spanned several sarcomeres (Figure 3B, right, red arrows) and arose more frequently from non-z-line locations (Figure S1C and S1D). Analysis of sbfSEM reconstructions showed that, compared with control, there was a trend toward fewer t-tubules in recovery, but their volumes were larger, in accordance with the increased tubule branching, resulting in complete restoration of t-tubule density (Figure 3D through 3G). This is the first report of such atrial t-tubule remodeling, and it is more extreme than that seen in the ventricle, for example^8,21^ We next sought to determine if t-tubule remodeling is associated with structural remodeling of other cellular components.

Normal atrial t-tubules extend along the z-line, between adjoining sarcomeres and mitochondria, toward the cell interior. We used confocal imaging of mitochondria (mito-tracker) and t-tubules (WGA) in recovered atrial myocytes to investigate if disordered atrial t-tubules were associated with mitochondrial disruption. Atrial mitochondria seem disrupted following recovery from HF, and confocal data (magnified region in Figure S2A) shows that disordered, recovered t-tubules (green) take an irregular path through mitochondria (red).

The precise relationship between disordered t-tubules and mitochondria was examined using sbfSEM (Figure 3H and 3I; Figure S2B). As expected, control atrial t-tubules extend between rows of interfibrillar mitochondria, which are 1 mitochondrion deep, but did not penetrate thicker mitochondrial bands (Figure 3H). Following recovery from HF, sbfSEM shows enlarged, that is, >1 mitochondrion-wide, interfibrillar mitochondrial bands with irregular arrangement, and large gaps occupied by debris (Figure 3I). Recovered t-tubules extended along the z-line in areas with single rows of interfibrillar mitochondria, like control (Figure 3I, left). However, in areas with widened mitochondrial bands, recovered t-tubules entered the available space (Figure 3I, middle), becoming highly branched and dilated, allowing the formation of oak-tree structures (Figure 3I, right). Our data suggest an association between t-tubule and mitochondria disruption, where disordered mitochondrial bands allow erratic t-tubule extension and displaced mitochondria divert normal t-tubule extension along the z-line, causing branching (Figure S2B). Despite this disorder, we next investigated whether recovered atrial t-tubules were associated with a restoration of the systolic Ca^2+^ transient.

### Systolic Ca^2+^ Transient Is Restored Following Recovery of the Atrial T-Tubule Network

**Figure 4.**
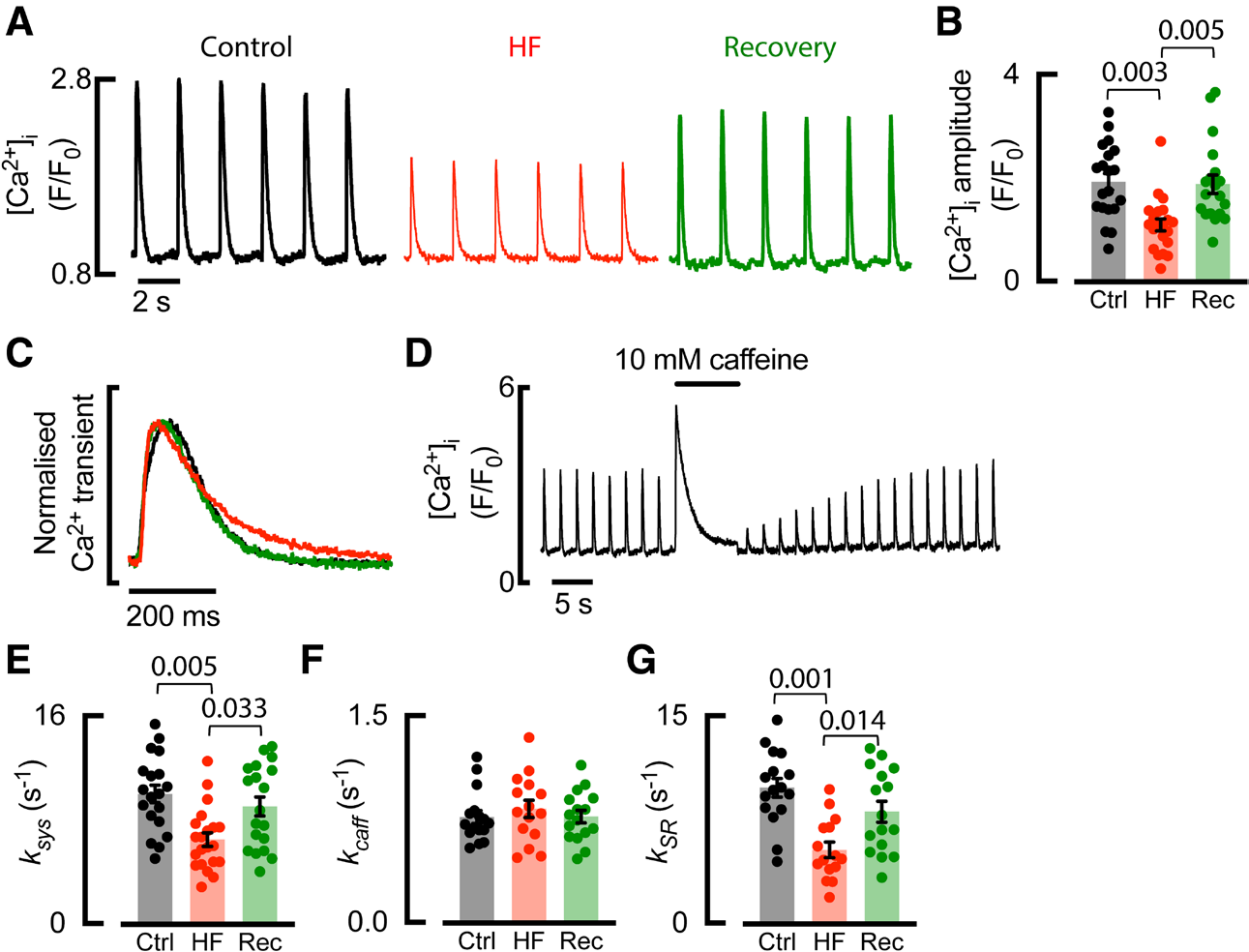
**Recovered t-tubules are associated with restored systolic Ca^2+^. A**, Representative Ca^2+^ transients and (**B**) systolic Ca^2+^ transient amplitude. **C**, Example normalized systolic Ca^2+^ transients. **D**, Typical experimental time course for Ca^2+^ including application of 10 mmol/L caffeine. **E**, Rate of decay of the systolic Ca^2+^ transient (_SYS_). **F**, Sarcolemmal (*k*_CAFF_) and (**G**) SERCA (*k*_SR_)-dependent rates of Ca^2+^ removal. Symbols denote cells n; control (Ctrl): n=20 (9 animals) for **B** and **E**, n*=*16 (8 animals) for **F** and **G**; heart failure (HF): n=21 (9 animals) for **B** and **E**, n*=*15 (8 animals) for **F** and **G**; recovery (Rec): n=19 (7 animals) for **B** and **E**, n*=*16 (6 animals) for **F** and **G**; compared using linear mixed modeling. Data are presented as mean±SEM.

Confirming our previous findings, epifluorescence recordings from voltage-clamped cells show the amplitude of the atrial systolic Ca^2+^ transient decreased in HF (Figure 4A and 4B).^5,19^ We now find that the restoration of functional t-tubules upon recovery is associated with restoration of the Ca^2+^ transient to control levels (Figure 4A and 4B). In addition, recovered atrial myocytes were able to respond appropriately to β-adrenergic stimulation (Figure S3).

When Ca^2+^ transients were normalized for size (Figure 4C), the slowed rate of decay of the Ca^2+^ transient (*k*_sys_) in HF was restored upon recovery (Figure 4C and 4E). To explore roles for *I*_NCX_ and SERCA in the restoration of Ca^2+^ transient decay, caffeine was applied (Figure 4D). The rate of decay of the caffeine-evoked Ca^2+^ transient (*k*_caff_), where SERCA is functionally blocked, was unchanged in HF and recovery. By subtracting *k*_caff_ (SERCA blocked) from *k*_sys_ (SERCA active), we calculated the rate of decay of the Ca^2+^ transient due to SERCA (*k*_SR_). We confirmed the previously described decrease in SERCA function in HF,^19^ and we show restoration of SERCA function upon recovery (Figure 4G). Therefore, our data suggest restored SERCA function underlies the recovery of the rate of decay of the Ca^2+^ transient. Ca^2+^ removal by routes other than SERCA and Na^+^-Ca^2+^ exchanger was unaltered (Supplemental Data), and so taken together, our data suggest *I*_NCX_ and *I*_PMCA_ were unchanged in HF and recovery. We next sought to determine whether disordered atrial t-tubules could serve as the site for initiating Ca^2+^ release in the cell, consistent with restoration of the Ca^2+^ transient.

### Despite Their Structural Disorder, Recovered Atrial T-Tubules Still Trigger Ca^2+^ Release

**Figure 5.**
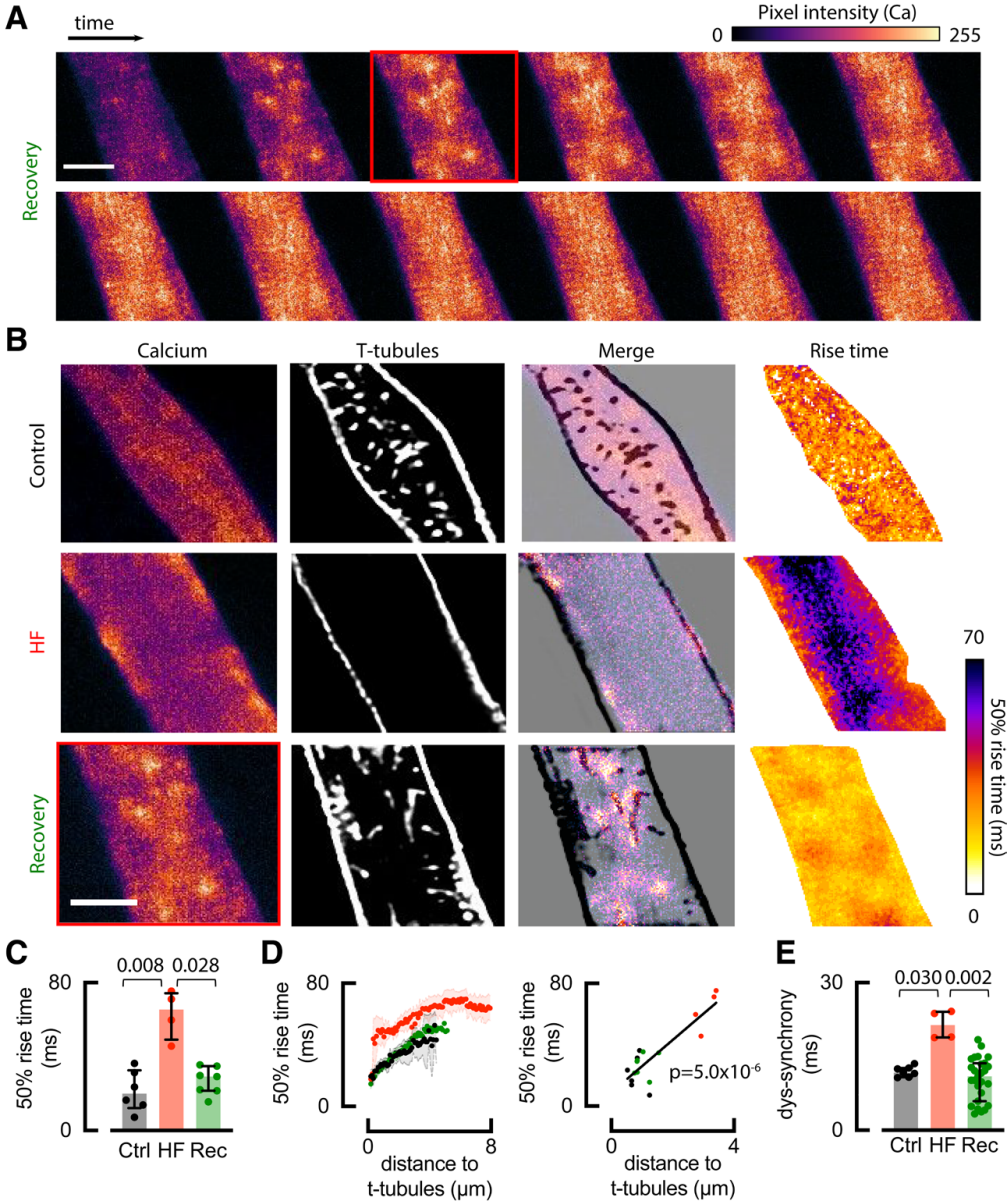
**Recovered t-tubules trigger Ca^2+^ release. A**, Representative time series from a recovery atrial cell showing early calcium release (fluorescence). **B**, Atrial myocytes showing; triggered calcium release, membrane staining, merge of calcium release, and membrane staining and Ca^2+^ rise time. **C**, Summary data for 50% Ca^2+^ rise time. **D**, Correlation between rise time and distance to t-tubules for all data points (left) and for t-tubule half distance (right, R^2^=0.7599). **E**, Dys-synchrony of Ca^2+^ release. Symbols denote cells n; control (Ctrl): n=6 (4 animals) for **C** and **D**, n*=*8 (2 animals) for **E**; heart failure (HF): n=4 (2 animals) for **C** to **E** (control and HF to confirm our previous work^5^); recovery (Rec): n=7 (3 animals) for **C** and **D**, n*=*27 (5 animals) for **E**; compared using Kruskal-Wallis test with multiple comparisons. Data are presented as median±interquartile range (IQR). Scale bars: 10 µm.

The rise in systolic Ca^2+^ was visualized in control, HF, and recovery cells using XY confocal imaging (Figure 5A shows an example recovery cell). [Ca^2+^]_i_ rose initially at discrete points and then spread, becoming uniform across the cell. The early rise of [Ca^2+^]_i_ was overlaid with the t-tubule/surface membrane staining (WGA) from the same confocal plane (Figure 5B). In control, systolic [Ca^2+^]_i_ rose first along the t-tubules, while in HF cells, lacking t-tubules, it was restricted to the cell surface as previously shown^5^ (Figure 5B). Following recovery, [Ca^2+^]_i_ initially rose along disordered t-tubules before propagating throughout the cell (Figure 5B), showing that new t-tubules enable triggered Ca^2+^ release in the cell interior.

Despite t-tubule disorder in recovery cells, the rate of rise of Ca^2+^ returned to control values, as opposed to the slowed central rise observed in HF (previously shown^5^ and confirmed in Figure 5B right and Figure 5C). The 50% rise time and distance to the nearest membrane (t-tubule or surface membrane) were calculated for each voxel, revealing that the rise time for Ca^2+^ increased with the distance of voxels from the membrane in control and recovery cells, where all voxels were <5.2 μm from the nearest membrane. In HF, this distance increased to 8 μm due to the loss of t-tubules (Figure 5D). Consistent with this, the dys-synchrony index was increased in HF but was restored to control levels when t-tubules returned following recovery from HF (Figure 5E). Our data suggest recovered t-tubules are as effective as control t-tubules in triggering Ca^2+^ release.

The effective triggering of Ca^2+^ release suggests the existence of functional dyads on newly formed atrial t-tubules. We next used a combination of immunocytochemistry and voltage clamp to investigate the localization and function of key dyadic proteins.

### Ordered Atrial Ryanodine Receptor Localization Is Lost in HF But Restored Following Recovery From HF

Triggered Ca^2+^ release requires dyads containing L-type Ca^2+^ channels and ryanodine receptors (RyRs). Immunocytochemistry revealed RyRs on the z-line in control and recovery, colocalizing with t-tubules (Figure S4A and S4B). Surface RyRs were sparse in control and recovery but increased in HF (Figure S4A bottom row and Figure S4B). RyR density does not increase in HF,^19^ suggesting in HF RyRs shift from the cell interior to the surface. Compared with control and recovery, where RyR staining appears continuous along the z-line, in HF RyR staining is fragmented between z-lines (Figure S4C). Fragmentation was measured as an increased number of structures along a sarcomere that could be resolved confocally (Figure S4D) and decreased order when RyR intensity was measured by a line perpendicular to the z-lines (Figure S4C, orange line and Figure S4E). We speculate that RyR fragmentation contributes to dys-synchronous Ca^2+^ release and RyR dispersal promotes Ca^2+^ wave propagation.^23^ The restoration of RyR localization upon recovery, at least at the confocal level, suggests RyR disorder is reversible and may contribute to decreased dys-synchrony upon recovery (Figure 5E). Recovered t-tubules, despite disorder, colocalize with RyRs where they bisect the z-line, presumably forming dyads for triggered Ca^2+^ release. Suitable L-type Ca^2+^ channel antibodies for sheep are currently unavailable, but the presence of Na^+^-Ca^2+^ exchanger on both normal (control) and disordered (recovered) atrial t-tubules supports the notion that recovered t-tubules associate with ion channels important for Ca^2+^ handling (Figure S4F and S4G). We next used voltage clamp experiments to determine if *I*_Ca-L_ density was restored to control levels following recovery, consistent with the formation of dyads at recovered t-tubules being more numerous than at the surface membrane.

### T-Tubule Recovery Is Associated With Restoration of Peak *I*_Ca-L_ and SR Ca^2+^ Content But Not Ca^2+^ Buffering

**Figure 6.**
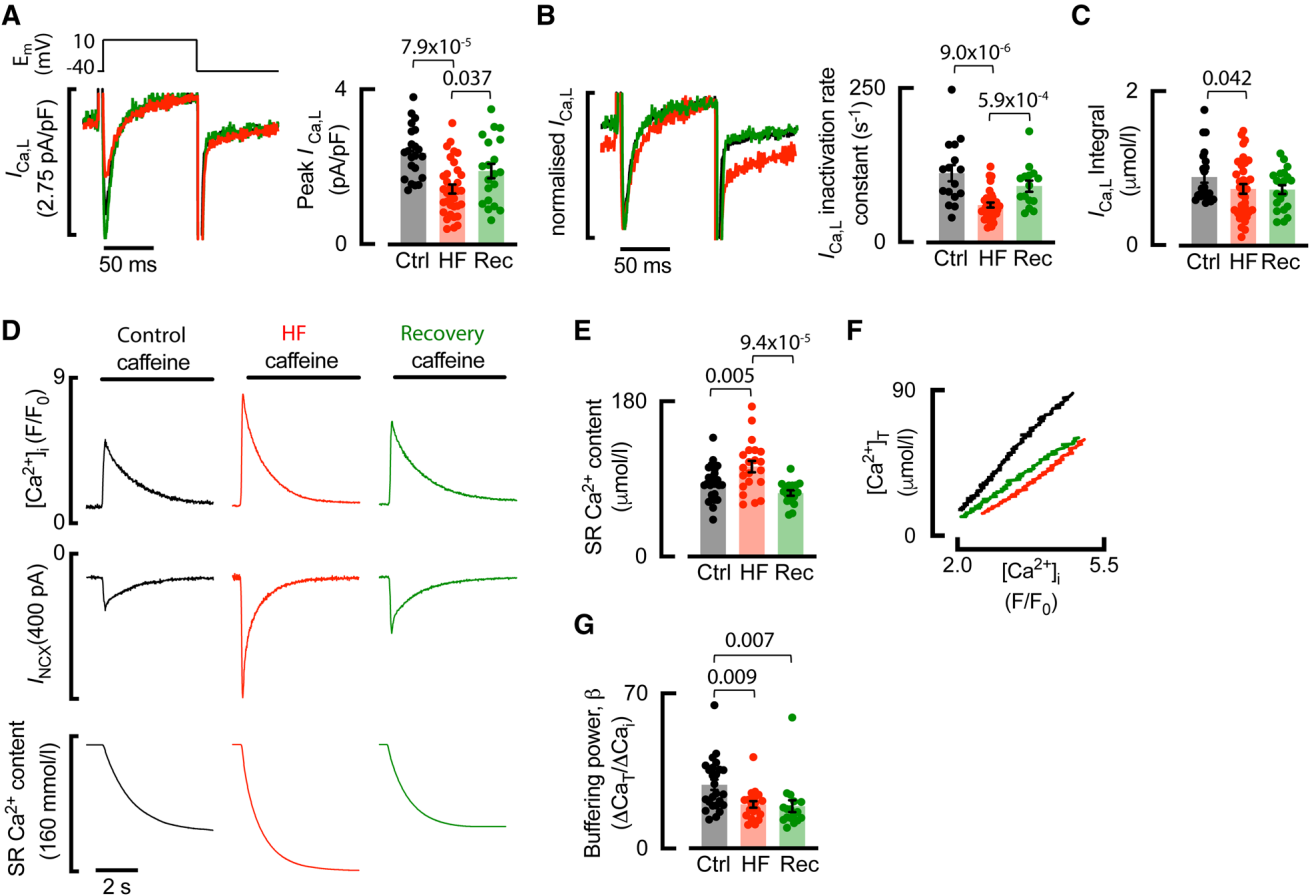
**Peak *I*_Ca-L_ and sarcoplasmic reticulum (SR) Ca content are restored following recovery of the atrial t-tubule network. A**, Example voltage step (upper) used to elicit *I*_Ca-L_ (lower) in control (Ctrl; black), heart failure (HF; red), and recovery (Rec; green) atrial myocytes, and data (right) for peak *I*_Ca-L_. **B**, Normalized *I*_Ca-L_ (left) and data (right) for the *I*_Ca-L_ inactivation rate constant. **C**, *I*_Ca-L_ integral. **D**, Quantitative assessment of free and total Ca in atrial myocytes. Caffeine-evoked free Ca^2+^ transients (top) are associated with inward *I*_NCX_ (middle), which was integrated in a cumulative manner and corrected for cell volume and Ca^2+^ removal via pathways other than Na^+^-Ca^2+^ exchanger (NCX) to give a measure of total Ca (lower). **E**, SR Ca content as calculated from **D**. **F**, Typical cellular buffer curves showing the relationship between free and total Ca. **G**, Cellular Ca^2+^ buffering power. Symbols denote cells n; control: n=22 (12 animals) for **A** and **C**; n*=*16 (11 animals) for **B**; n*=*25 (16 animals) for **E** and **G**; HF: n=35 (14 animals) for **A** to **C**, n=21 (12 animals) for **E** and **G**; recovery: n=21 (6 animals) for **A** and **C**, n*=*15 (6 animals) for **B**; n*=*17 (6 animals) for **E** and **G**; compared using linear mixed modeling or simple linear regression. Data are presented as mean±SEM.

Supporting the notion of preferential dyad formation at the recovered t-tubule, the decreased peak *I*_Ca-L_ density associated with t-tubule loss in HF^19^ increased to control levels upon recovery from HF (Figure 6A). Consistent with L-type Ca^2+^ channels residing in a dyad with close coupling to SR Ca^2+^ release and subsequent Ca^2+^ dependent inactivation, the slowed rate constant of *I*_Ca-L_ inactivation in HF was restored to control levels following recovery of t-tubules (Figure 6B). The integrated Ca^2+^ entry via *I*_Ca-L_ was not restored upon recovery (Figure 6C), likely because although peak and inactivation of recovered *I*_Ca-L_ were not different to control, numerical values did not recover completely.

Given that we previously demonstrated that reduced peak *I*_Ca-L_ is responsible for elevated atrial SR Ca^2+^ content in HF,^19,24^ we next aimed to clarify if restoration of peak *I*_Ca-L_ during recovery could bring SR Ca^2+^ content back down to control levels. SR Ca^2+^ content was measured from the integral of the caffeine-evoked Na^+^-Ca^2+^ exchanger current^25^ (Figure 6D). We found that the increased SR Ca^2+^ content in HF was restored back to control levels following recovery from HF (Figure 6E).

Finally, we used experiments similar to Figure 6D to assess the Ca^2+^ buffering capacity of the cell by examining the relationship between total and free Ca^2+^ (Figure 6F). We confirmed the decrease in Ca^2+^ buffering in atrial cells in HF, whereby the relationship between free and total Ca^2+^ was shallower (Figure 6F) and the Ca^2+^ buffer power (the ratio of change of total to free Ca^2+^) was decreased (Figure 6G). We found that, in contrast to peak *I*_Ca-L_ and SR Ca^2+^ content, the decreased Ca^2+^ buffering in HF was not restored upon recovery (Figure 6F and 6G) and may, therefore, facilitate recovery of the Ca^2+^ transient amplitude.

Our data suggest recovered t-tubules play an important role in the restoration of the Ca^2+^ transient. Our next experiments were designed to precisely determine their specific contribution.

### Acute Removal of Recovered T-Tubules Converts the Recovery Ca^2+^ Transient Back to a HF-Like Phenotype

**Figure 7.**
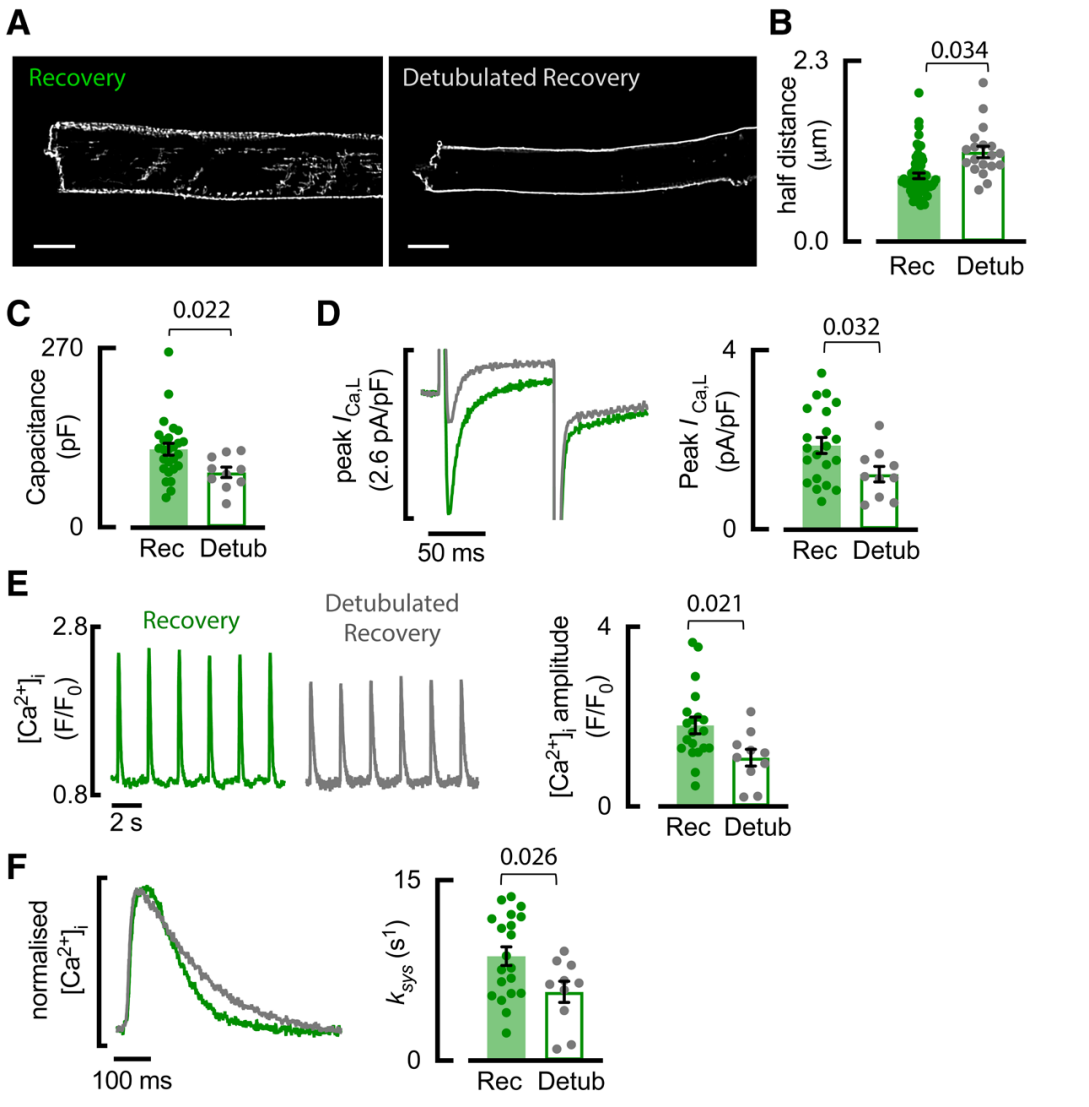
**Detubulation (Detub) of recovered atrial myocytes converts the recovery Ca^2+^ transient back to a heart failure (HF)-like phenotype. A**, Di-4-ANEPPS staining of recovery sheep atrial myocytes showing t-tubule loss following detubulation. Scale bars: 10 µm. **B**, Half distance to nearest t-tubule and surface membrane. **C**, Cellular capacitance of recovery atrial myocytes. **D**, Representative and mean data for peak *I*_Ca-L_ in recovery (Rec; gray) and detubulated (Detub) Rec (gray) atrial myocytes. **E**, Representative systolic Ca^2+^ transients and mean systolic Ca^2+^ transient amplitude. **F**, Example normalized systolic Ca^2+^ transients, and mean rate of decay of the systolic Ca^2+^ transient (*k*_SYS_). Symbols denote cells n; Rec n=61 (6 animals) for **B**, n=26 (7 animals) for **C**, n=20 (6 animals) for **D** to **F**; Detub n=19 (3 animals) for **B**, n=10 (4 animals) for **C** to **F**; compared using linear mixed modeling. Data are presented as mean±SEM.

The importance of recovered t-tubules was directly established by using acute detubulation with formamide,^26^ such that they uncoupled from the surface membrane and were no longer accessible to our membrane dye (Figure 7A). As expected, the removal of recovered t-tubules increased the distance of points in the cell interior to the membrane (Figure 7B), and patch clamp revealed a decrease in cellular capacitance consistent with membrane loss (Figure 7C). The removal of recovered t-tubules decreased peak *I*_Ca-L_ (Figure 7D), the amplitude of the systolic Ca^2+^ transient (Figure 7E), and the rate constant of decay of the Ca^2+^ transient (*k*_sys_; Figure 7F). All 3 parameters reverted back to HF levels (Figure S5), suggesting that the recovery of disordered t-tubules was directly responsible for the recovery of these parameters.

Given the clear importance of restored t-tubules to the recovery of Ca^2+^ handling, we next set out to determine the factors responsible for building new t-tubules.

### Increased Myotubularin in the Recovery Atria May Underlie Increases in T-Tubule Density, Length, and Branching

**Figure 8.**
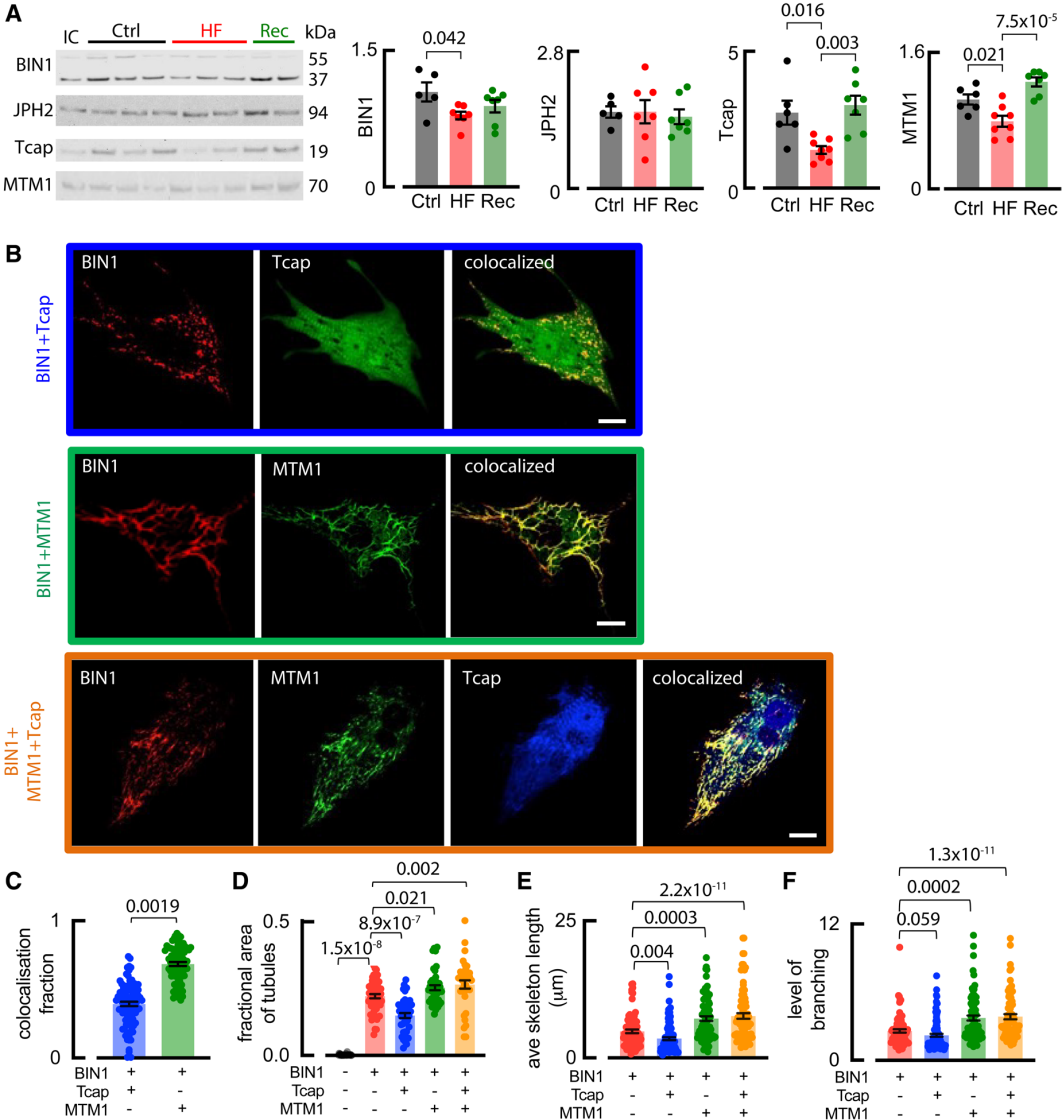
**MTM1 (myotubularin 1) and Tcap (telethonin) influence t-tubule density and structure. A**, Western blots and mean data for BIN1 (bridging integrator 1/amphiphysin II), Tcap, MTM1, JPH2 (junctophilin 2) in control (Ctrl), heart failure (HF), and recovery (Rec) sheep atrial tissue. Symbols denote animals N for **A**, Control: N=5 for BIN1 and JPH2, N=6 for Tcap and MTM1; HF: N=7 for BIN1 and JPH2, N=8 for Tcap and MTM1; recovery: N=7 for BIN1, JPH2, Tcap, and MTM1, compared using ANOVA with multiple comparisons. **B**, NRVMs transfected with BIN1, Tcap, or MTM1 or triple transfection of BIN, MTM1, and Tcap. **C**, Fraction of colocalization with BIN1. Mean data summarizing: (**D**) fractional area of cells occupied by tubules; (**E**) average skeleton length; and (**F**) average branching of each tubule structure. Symbols denote cells n for **C** to **F**; Ctrl vector n=45 cells (5 L); BIN1: n=79 cells (7 L); BIN1+Tcap: n=98 cells (8 L); BIN1+MTM1: n=84 cells (6 litters); and triple transfection: n=77 cells (5 L); compared using linear mixed modeling. Data are presented as mean±SEM. Scale bars: 10 µm.

Previous work has identified proteins involved in ventricular t-tubule formation. We have, therefore, investigated how their abundance changed during the development and recovery from HF in the atrium. Figure 8A shows that BIN1 decreased modestly in HF but upon recovery was not different to control or HF, suggesting other proteins contribute to new t-tubule growth. JPH2 exhibited no measurable change, while Tcap, a load-dependent regulator of ventricular t-tubules,^10,14,18^ correlated with atrial t-tubule abundance (Figure 8A). Additionally, MTM1 (myotubularin), a BIN1 binding partner, and regulator^27^ correlated with atrial t-tubule density (Figure 8A).

To assess the causal involvement of Tcap and MTM1 in t-tubule recovery, we investigated whether increased expression of these proteins, as observed in recovery from HF, directly alters t-tubule density or structure using neonatal rat ventricular myocytes, which lack t-tubules. We found that expression of the control vector, MTM1 or Tcap alone, failed to produce tubules, whereas (as we have shown previously^14^) expression of BIN1 did (Figure S6A). BIN1-driven tubules were connected to the extracellular environment as shown by extracellular dye penetration (Oregon Green), which colocalized with BIN1 (Figure S6B). We coexpressed BIN1 with either Tcap, MTM1, or both to elucidate any role of these in modulating BIN1 tubulation (Figure 8B). Coexpressing BIN1 with either Tcap, MTM1, or both revealed that MTM1 colocalized with BIN1 at tubules, while little colocalization occurred between BIN1 and Tcap (Figure 8B and 8C). Compared with BIN1 alone, tubule density decreased with Tcap coexpression but increased with MTM1 coexpression (Figure 8D).

Mimicking recovery from HF, the triple transfection (BIN1+MTM1+Tcap) increased t-tubule density compared with BIN1 alone, suggesting the dominant effect of MTM1 over Tcap and implicating MTM1 in increased atrial t-tubule density in post-HF recovery. Given the longer, more branched t-tubules in recovery atria, we investigated whether either protein could alter the structure of BIN1-driven tubules. Both average skeleton length (equivalent to the length of a single tubule structure) and branching decreased with Tcap but increased with MTM1, indicating both proteins can modulate tubule structure (Figure 8E and 8F). Again, the MTM1 effect dominated in the triple transfection for both parameters, suggesting that increased MTM1 plays a role in the enhanced atrial t-tubule length and branching following recovery from HF. In summary, our data demonstrate that increased MTM1, or MTM1+Tcap, results in longer and more branched BIN1-driven tubules, reminiscent of the increased t-tubule length and branching observed in recovery atrial cells (Figure 1) with elevated MTM1 and Tcap levels.

## DISCUSSION

In this study, we show atrial t-tubules can be restored following their loss in HF, and this restores the systolic Ca^2+^ transient; we also provide a mechanistic understanding of t-tubule and Ca^2+^ handling restoration. Specifically we show (1) complete restoration of atrial t-tubule density by fewer, but longer and more branched t-tubules; (2) recovered t-tubules are disordered and associated with mitochondrial disorder; (3) recovered t-tubules trigger Ca^2+^ release and underlie restoration of peak *I*_Ca-L_, which, together with the restoration of RyR structure, subsequently restores SR Ca^2+^ content; (4) t-tubule recovery is responsible for restoring the systolic Ca^2+^ transient and synchrony of Ca^2+^ release; and (5) Tcap and MTM1 influence t-tubule density and structure, with MTM1 likely driving the re-growth of longer, more branched t-tubules following recovery from HF. These findings highlight the reversibility of t-tubule loss, RyR remodeling, and Ca^2+^ handling alterations in HF, providing a promising therapeutic target for future studies targeting contractile dysfunction in HF.

### Atrial T-Tubules Can Recover Following Their Almost Complete Loss in HF

In HF, t-tubule loss from atrial and ventricular myocytes^2,5,28,29^ contributes to decreased contractility,^4,30^ with the almost complete loss of atrial t-tubules being the most severe.^8^ In the present study, we demonstrate for the first time the synthesis of new atrial t-tubules, formed following tachypacing termination, which restores t-tubule density to control levels. While interventions aimed at restoring cardiac function have increased ventricular t-tubule density or prevented t-tubule loss, in the failing ventricle,^9–11,14,15^ distinguishing recovered ventricular t-tubules from preexisting t-tubules is challenging. The atria provide a unique opportunity to explore the functional impact of restored t-tubules on Ca^2+^ release, necessary for potential therapeutic interventions.

The mechanisms of t-tubule loss and recovery in disease are unknown, although cardiac load appears important.^9,31–33^ Evidence suggests early t-tubule remodeling may contribute to disease progression,^3^ highlighting the potential of early interventions to restore t-tubules and preserve cardiac function. Support for this concept comes from the beneficial effects observed following cardiac unloading in patients with HF before excessive remodeling.^33^ In addition, our work suggests intervention can be beneficial even after substantial remodeling, as demonstrated in the current study, and in the presence of rapid ventricular pacing, where phosphodiesterase-5 inhibition restored t-tubules and improved contraction.^14^ Therefore, t-tubule recovery and improved contraction are possible, offering potential benefits even as HF progresses.

### Recovered Atrial T-Tubules Are Disorganized

Despite the functional importance of atrial t-tubules, little is known about their ultrastructure. As expected, we find normal sheep atrial t-tubules are predominantly transverse, occurring at z-lines, a pattern observed in human atrial tissue,^6^ but in contrast to smaller mammals, where most (not all^34^) studies report that t-tubules are absent or sparse and irregular.^26,35–39^ Unlike the relatively uniform ultrastructure of ventricular t-tubules,^21^ normal atrial t-tubules have diverse morphologies. The function of this diversity is unknown, but local increases in t-tubule diameter in structures such as the club may increase dyad area,^40^ potentially supporting Ca^2+^ release.

Following recovery from HF, major changes are seen in the ultrastructure and arrangement of newly formed t-tubules, presenting in pairs, and complex, highly branched, and disordered structures with both transverse and longitudinal elements. Such disorder has not previously been reported in the adult atria or following reverse ventricular remodeling, where interventions to treat HF improve t-tubule organization.^9–13,15^ However, in HF itself, ventricular t-tubules may become disordered, the most extreme examples being t-tubule sheets.^33,41,42^ This level of disorder would make identification of any newly formed ventricular t-tubules unreliable (if their structure was similar to recovered atrial t-tubules). The atrial t-tubule disorder we report was, however, distinct specifically to t-tubule sheets; recovered atrial t-tubules were highly branched in all directions, often extended longitudinally through the cell (eg, oak tree, longitudinal, and lattice), and could deviate from the z-line. In contrast, t-tubule sheets are widened in a single plane parallel to the myocyte and maintain their transverse orientation.

Ventricular studies, for example, in human,^41^ show increased longitudinal t-tubules in HF. In other scenarios, longitudinal t-tubules have been associated with mechanisms to increase systolic Ca^2+^, for example, phosphorylated RyRs^35^ or increased Na^+^-Ca^2+^ exchanger-generated Ca^2+^ influx,^43^ and therefore they may be compensatory in HF. Newly formed atrial longitudinal t-tubules may facilitate the rise in Ca^2+^ by similar mechanisms; however, their extreme disorder is inconsistent with efficient dyad formation since t-tubules and RyRs only colocalize at discrete points. Therefore, this disorder in atrial t-tubules may, in part, be a consequence of cellular structural disorder remaining from HF.

Cellular structural remodeling in HF provides a distinct platform for t-tubule formation. A striking alteration was mitochondrial reorganization, which has not previously been linked with t-tubule structure. In the ventricle, HF induces mitochondrial damage and displacement,^44,45^ and disorder is also present in atrial fibrillation (for review, see ^46^). Although we cannot confirm if mitochondrial remodeling precedes or follows new t-tubule formation, our data align with the idea that widened, less tightly packed bands of mitochondria allow t-tubule formation in the available space upon recovery from HF. Therefore, mitochondrial disorder may contribute to disturbed t-tubule structure.

### Post-HF Recovery of Atrial T-Tubules Restores *I*_Ca-L_, the SR Ca^2+^ Content, and Triggered Ca^2+^ Release Which, Together With Recovery of RyR Localization, Restores Systolic Ca^2+^

Atrial t-tubule loss in atrial fibrillation^7^ and HF^5^ is associated with decreased *I*_Ca-L_, which our earlier studies suggest decreases the atrial systolic Ca^2+^ transient.^19^ The finding that systolic Ca^2+^ rose first along t-tubules in recovery atrial cells suggests these disordered t-tubules form dyads. This is consistent with our detubulation data showing recovered t-tubules are responsible for restoration of the Ca^2+^ transient amplitude, the rate of decay of the Ca^2+^ transient, and peak *I*_Ca-L_, emphasizing that recovered *I*_Ca-L_ resides on recovered t-tubules. This suggests the cellular processes responsible for *I*_Ca-L_ turnover and t-tubule localization^47^ are restored following recovery from HF. The fact that recovered *I*_Ca-L_ inactivation is t-tubule dependent is likely due to restoration of the spatial characteristics of Ca^2+^ release and recovery of Ca^2+^ dependent inactivation in-line with triggered, dyadic Ca^2+^ release. Similarly, the dependence of Ca^2+^ transient decay on t-tubules could be explained by t-tubules promoting central Ca^2+^ release in the proximity of SERCA, given we found no change in the abundance of SERCA or phospholamban in HF,^19^ highlighting the importance of the spatial restoration of Ca^2+^ release.

Disordered RyR structure is thought to contribute to perturbations of Ca^2+^ release in disease.^23,48^ This is consistent with the fragmentation of RyR Z-line staining in HF, which could impede Ca^2+^ propagation, suggesting restored RyR structure contributes to the recovered Ca^2+^ transient synchronicity and amplitude. Increased RyR colocalization with the cell surface in HF may compensate for t-tubule loss by facilitating surface Ca^2+^ release, similar to enhanced surface Ca^2+^ release in neonatal sheep atrial cells with sparse t-tubules.^49^ How RyR redistribution occurs in this study is unknown, but decreased RyR abundance (as reported in the atria in HF^50^) alters RyR cluster structure,^51^ as does CAMKII,^52^ which is increased in the sheep atria in HF.^19^ Thus, these factors may play a role in the altered RyR distribution we report.

Our study is the first, to our knowledge, to show restoration of atrial RyR organization at the confocal level following disruption in HF, providing another mechanism to improve dyad function.

We have previously shown that decreased *I*_Ca-L_ underlies increased atrial SR Ca^2+^ content in HF and aging.^19,24^ Therefore, here we suggest t-tubule-dependent *I*_Ca-L_ restoration is responsible for the decrease in SR Ca^2+^ content to control levels following recovery from HF. Thus, t-tubule recovery restores *I*_Ca-L_ and SR Ca^2+^ content, the 2 main factors governing Ca^2+^ transient amplitude.^53^

Since we show it is possible to restore RyR structure, in addition to *I*_Ca-L_ and SR Ca^2+^ content, it may be surprising that t-tubule recovery and the restoration of *I*_Ca-L_ and SR Ca^2+^ content are sufficient to fully account for recovery of the Ca^2+^ transient amplitude. Two factors are important to consider here, which may increase the Ca^2+^ transient in HF. First, increased surface RyRs in HF would facilitate systolic Ca^2+^ and was not accounted for during detubulation. Second, in HF, the decrease in Ca^2+^ buffering would promote the systolic Ca^2+^ transient.^24^ Interestingly, this decrease in Ca^2+^ buffering persists following recovery, but its importance to Ca^2+^ propagation in recovery versus HF, where t-tubules are lacking, may be decreased, and this difference could mask any RyR structural effect.

The mechanism underlying the decrease in Ca^2+^ buffering in HF and recovery remains unknown. Given the main cytosolic Ca^2+^ buffers are SERCA and the myofilaments, and that the decay of the Ca^2+^ transient (largely due to SERCA function) is restored upon recovery, our data point to a role for the myofilaments. A decrease in troponin’s affinity for Ca^2+^ could explain the decrease in Ca^2+^ buffering at low [Ca^2+^]; this would necessitate higher Ca^2+^ levels for myofilament activation with detrimental effects on contraction.

The extent to which atrial t-tubules contribute to the systolic Ca^2+^ transient is an important question. Compared with the ventricle, atrial cells have fewer t-tubules and Ca^2+^ rises due to both triggered Ca^2+^ release at membrane sites and propagation to sites where t-tubules are lacking. It is therefore reasonable to assume that atrial t-tubules contribute less to the Ca^2+^ transient than those in the ventricle. However, changes in factors other than t-tubules in HF, principally Ca^2+^ buffering, SR Ca^2+^ content, and RyR arrangement, complicate such direct assessments. On the contrary, acute detubulation of recovered atrial myocytes clearly shows the importance of recovered t-tubules, although their structural disorder may make them less efficient than control atrial t-tubules.

Taken together, we suggest recovered atrial t-tubules and improved RyR organization restored triggered Ca^2+^ release in the cell interior, improving synchronicity, and restoring the Ca^2+^ transient amplitude.

### Recovery of Proteins Associated With T-Tubule Biogenesis

We found that atrial t-tubule restoration was associated with increased BIN1, Tcap, and MTM1, but not JPH2. While JPH2 has been linked to t-tubule organization and HF-associated t-tubule loss in rodents, data suggest its lack of involvement in t-tubule loss or recovery in our sheep model of HF and retubulation in some ventricular models.^3,8–10,32,54^ Conversely, BIN1 overexpression drives tubule formation^14,17,55,56^ in various cell types lacking t-tubules, and its expression correlates with t-tubule density.^8,10,14^ We show Tcap, in agreement with ventricular studies, is also correlated with atrial t-tubule density, indicating its role as a load-sensitive t-tubule regulator.^9,18^

In skeletal muscle, MTM1 enhances BIN1-mediated membrane tubulation, t-tubule structure, and Ca^2+^ handling.^27,57^ The decrease in L-type Ca^2+^ current and impaired SR Ca^2+^ release observed in this study is consistent with previous observations in skeletal muscle lacking MTM1.^58,59^ MTM1 coexpression in skeletal muscle led to longer, more reticulated BIN1-driven t-tubules.^27^ This is consistent with our study, showing that when MTM1 levels are increased, in either recovery or neonatal rat ventricular myocyte overexpression, t-tubules are longer and more branched.

Unlike MTM1, Tcap overexpression suppressed BIN1-mediated tubulation. Importantly, recovery from HF is associated with increased Tcap and MTM1, and our data show the MTM1 effect predominated, increasing t-tubules. Our findings demonstrate the role of MTM1 in modulating cardiac t-tubule density and structure. We suggest that increased MTM1 in the recovered atria is implicated in enhanced t-tubule density, length, and branching, shedding light on the significance of MTM1 in cardiac function.

### Comparison With Ventricular T-Tubules

While understanding the mechanisms of atrial remodeling in disease is of fundamental importance in its own right, it is also of value to consider if the same processes operate in the ventricle. T-tubule formation and turnover depend on key players such as BIN1, Tcap, and MTM1. However, the importance of these is likely differentially regulated by protein abundance or localization and, importantly, by differential pathological stressors such as stretch, which may vary between species and the chambers of the heart.

The splicing pattern of BIN1 is species–specific,^55^ and multiple splice variants form t-tubules, with different properties.^55,60^ However, a key difference between variants is the phosphoinositide binding domain, which is necessary for MTM1 binding.^27,60^ Variants containing the phosphoinositide domain are more potent regulators of t-tubules, and while present in sheep and humans, they are absent in the mouse.^14,17,55,60^ When MTM1 is overexpressed, the presence of the phosphoinositide domain in our study likely explains the MTM1:BIN1 colocalization and may be important for the increased t-tubule length and branching, which is absent in the mouse ventricle lacking the phosphoinositide domain.^60^ Thus, we suggest that, at least when the phosphoinositide domain is present, MTM1 is important for t-tubule restoration in addition to its role in t-tubule development.^60^ This supports the idea that specific t-tubule associated proteins are important between species but could perform subtlety different roles in different species.

The decrease of MTM1 abundance in HF is not observed in the ventricle.^14^ In HF, atrial dilatation presumably results from atrial stretch in response to ventricular dysfunction. We suggest stretch is more relevant to the thin-walled atria, where Laplace’s law suggests that upon a given increase in pressure, the thin-walled atria would experience higher wall stress, and therefore we presume increased stretch. This is consistent with a decrease in the stretch-sensitive protein Tcap^18^ in the atria (present study) but not in the ventricle,^14^ where we speculate stretch is less important. This supports the concept that pathways modulating t-tubules may be differentially affected by the different levels of stressors between chambers, which in turn could affect t-tubule abundance.

Taken together, atrial t-tubule loss is an important driver of dysfunction in pathology^7^ and worthy of understanding. The almost complete loss of atrial t-tubules in HF also provides us with a translationally relevant model to study t-tubule recovery. Evidence suggests the insight we provide is likely relevant to humans (in terms of BIN1 variants) and is not atrial-specific, having relevance to atrial and ventricular chambers.

### Conclusions

We have demonstrated atrial t-tubule restoration in sheep following near-complete loss in HF. Restored t-tubules were functional, triggering central Ca release and restoring the L-type Ca^2+^ current and the systolic Ca^2+^ transient. Our data suggests MTM1 regulates t-tubule density and structure in the heart and is important for the restoration of atrial t-tubules we observed following recovery from HF. Our model has provided unique insights into restored t-tubule structure and function and has advanced our understanding of the mechanisms that control t-tubule recovery. Thus, this research identifies several targets for the development of a therapeutic strategy for t-tubule restoration following loss in disease, most notably MTM1.

## ARTICLE INFORMATION

### Acknowledgments

The authors thank the staff in the Faculty of Biology, Medicine and Health Electron Microscopy (EM) Core Facility for their assistance and the Wellcome Trust for equipment grant support to the EM Facility. Graphical abstract was created using BioRender.com.

### Sources of Funding

This study was supported by research grants from The British Heart Foundation (FS/12/34/29565, PG12/89/29970, FS/14/4/30532, PG/18/24/33608, PG/15/70/31724, IG/15/2/31514, FS/17/54/33126, and PG/19/63/34601).

### Disclosures

None.

### Supplemental Material

Expanded Materials and Methods

Tables S1 and S2

Figures S1–S6

## Supplementary Material


