# Supplementary material for: Restoring Atrial T-Tubules Augments Systolic Ca Upon Recovery From Heart Failure
Source: Circ Res. 2024 Aug 14;135(7):739–54. doi: 10.1161/CIRCRESAHA.124.324601 (PMC11392124; doi:10.1161/CIRCRESAHA.124.324601)
Supplement: Supplementary file 2 [file res-135-739-s002.pdf]

Supplemental Material

Restoring atrial t-tubules augments systolic Ca upon recovery from heart failure

Jessica L. Caldwell#, Jessica D. Clarke#, Charlotte E.R. Smith, Christian Pinali, Callum J. Quinn, Charles M. Pearman, Aiste Adamoviciene, Emma J. Radcliffe, Amy E. Watkins, Margaux A Horn, Elizabeth F. Bode, George W.P. Madders, Mark Eisner, David A. Eisner, Andrew W. Trafford & Katharine M. Dibb*

Unit of Cardiac Physiology
Manchester Academic Health Science Centre
University of Manchester
M139NT
UK.

*Corresponding Author

# Contributed equally

Katharine.Dibb@manchester.ac.uk

Tel+44-161-275-1195

Methods

All procedures involving animals accord to the United Kingdom (Scientific Procedures) Act of 1986 and the University of Manchester Ethical Review Board.

Tachypacing induced heart failure in the sheep – Welsh Mountain sheep were provided by the Biological Services Facility, The University of Manchester. All sheep used in this study were female as it was not possible to obtain sufficient numbers of adult male sheep. Power calculations were performed, after determination of between and within subject variance, based on preliminary data or previous publications. Group sizes are given in figure legends and were determined using $\alpha = 0.05$ and $1\text{-}\beta = 0.8$. 63 female sheep (~ 18 months of age) were randomly assigned to control (non-instrumented), heart failure (HF) or recovery groups. Under isoflurane anaesthesia (1–4% v/v in oxygen) 36 sheep were instrumented with a pacing lead (St Jude Medical or Medtronic), fixed transvenously at the right ventricle apex and attached to a subcutaneous cardiac pacemaker (Medtronic). Sheep were given post-operative analgesia (meloxicam 0.5 mg/kg) and antibiosis (enrofloxacin 2.5 mg/kg). Animals recovered post-operatively for one week before tachypacing was initiated. HF was induced by rapid ventricular pacing (210bpm; median 35 days, interquartile range 12.5 days) as described previously.[5, 19] At the point of HF pacing was terminated in a sub-set of sheep that were allowed to recover for 37.±1.2 days. Animals were monitored daily for onset of clinical signs of HF (lethargy, dyspnoea and weight loss) and cardiac function was assessed regularly in conscious animals by trans-thoracic echocardiography as described previously.[8, 19]

Isolation of sheep atrial cardiac myocytes - Sheep were killed (heparin 10,000 units and pentobarbitone 200 mg kg^{-1} intravenously), the heart excised and the ventricles and atria were separated. The left circumflex coronary artery of the left atria was cannulated and left atrial myocytes isolated using a collagenase and protease digestion technique as described previously.[5, 19, 24] Atrial cells were stored in a normal Tyrode solution containing (in mM): 140 NaCl, 10 glucose, 10 Hepes, 4 KCl, 1.8 $CaCl_2$, 1 $MgCl_2$ and 2 probenecid (pH 7.34 with NaOH).

Cellular electrophysiology, measurement of intracellular Ca^{2+}, SR Ca^{2+} content and Ca^{2+} buffering – In previous work, we have studied the effects of heart failure on atrial myocytes. In order to reduce animal usage, we have included data from these animals as well as performing new experiments. The data from previous work comprises 13 cells from 22 and 21 from 35 cells for control and HF $I_{Ca\text{-}L}$ recordings, 11 from 20 and 21 from 30 control and HF Ca^{2+} transient recordings and 11 from 17 and 17 from 21 control and HF SR Ca^{2+} content recordings have been previously published [19] or [24]). All other data, including all recovery data is novel. Left atrial myocytes were loaded with Fluo-5F AM (5 µmol.l^{-1}; F14222, Molecular Probes) for 10 minutes to measure intracellular Ca^{2+} ($[Ca^{2+}]_i$). Following de-esterification (>45 minutes) myocytes were excited at 488nm, emitted light was captured at >515 nm and $[Ca^{2+}]_i$ calculated using the equation:

$$[Ca^{2+}]_i = k_d \frac{(F - F_{min})}{(F_{max} - F)}$$

Where, at a given wavelength, k_d is the dissociation constant of Fluo-5F at 37°C (1035 nmol l^{-1}), F is the florescence, F_{min} the florescence in the absence of Ca^{2+} (which is 0 for Fluo-5F;) and F_{max} is the florescence in the presence of saturating Ca^{2+} and was obtained at the end of the experiment.[19, 24] Intracellular Ca^{2+} was measured simultaneously with membrane currents.[19, 24] Perforated patch clamp with amphotericin-B (240 µg ml^{-1}) was used to achieve voltage clamp control of the cell with switch clamp applied at a frequency of 3-5 kHz to overcome access

resistance (Axoclamp-2B voltage clamp amplifier). Micropipettes with a resistance of 2-3 MΩ were filled with (in mM): 125 KCH_3O_3S, 20 KCl, 10 NaCl, 10 Hepes and 5 $MgCl_2$ titrated to pH 7.2 with KOH. $BaCl_2$ (0.1 mM), 4-aminopyradine (5 mM) and DIDs (0.1 mM) were used to block any contaminating K^+ or Cl^- currents during recordings. $I_{Ca\text{-}L}$ was elicited using a 100ms depolarizing step from -40 mV to + 10 mV.[19]

The surface area to volume ratio of control, HF and recovery cells was calculated from calcein-AM loaded cells as we have done previously.[19] Resultant values of 5.05, 4.89 and 4.71 pF pl^{-1} were used for control, HF and recovery cells respectively. The SR Ca^{2+} content was determined by rapidly applying 10 mM caffeine and integrating the resultant NCX current relative to the total cell volume (obtained from cell capacitance measurements and the surface area, volume relationship). The NCX integral was corrected for Ca^{2+} removal by other, non-electrogenic, pathways using a correction factor calculated in a separate series of experiments (as we have described previously. [24] The resulting correction factor was not different between control, HF and recovery cells and therefore a common correction factor of 1.2 was used in this study.

Cellular Ca^{2+} buffering was measured as described previously.[24] In brief, the corrected NCX integral during caffeine application gives a measure of the change in total intracellular Ca^{2+} (free Ca^{2+} + Ca^{2+} bound to intracellular buffers) but Fluo-5F gives a measure of free intracellular Ca^{2+}. The relationship between free and total Ca^{2+} give the buffering power of the cell.[19]

Formamide induced osmotic shock and detubulation – t-tubules were acutely removed from recovery atrial cells using osmotic shock.[26] Formamide (1.5 mol l^{-1}) was applied to the bathing solution for 15 minutes before rapidly returning cells to the control solution. A sub-set of recovery and detubulated recovery cells were examined confocally with di-4-ANEPPS (D1199, Molecular Probes, ThermoFisher) (as described below) to confirm successful detubulaton.

Isolation of neonatal rat ventricular myocytes, cell culture and transfection - Ten litters (total) of two-day old Wistar rats (Charles River UK ltd) were killed by cervical dislocation and decapitation. Bodies were rinsed in 70% ethanol and hearts excised and placed in ice cold disassociation buffer (containing (mM): 116 NaCl; 5.6 glucose; 20 HEPES; 5.4 KCl; 0.83 $MgSO_4$; 1 NaH_2PO_4; (pH 7.35)). Single ventricular myocytes were isolated using warmed dissociation buffer, containing 0.75mg/ml Collagenase A (500 ug, 0.21 U/mg, 0103586, Roche), and 1.3mg/ml Pancreatin (P-3292, Sigma), stirred at 120 rpm on a magnetic stirrer plate at 37°C for 7 minutes followed by titration. Serial digestions were performed until the ventricles were completely digested. Myocytes were re-suspended in pre-plating media (containing; 68% DMEM, 17% Medium 199 (M199) (22350-029, Life Tech,), 5% FBS, 1% fungizone, 1% penicillin streptomycin (penstrep; 10,000 units Pen/10mg/ml Strep) (Gibco, Life Technologies) and 10% horse serum (H1138, Sigma), plated on tissue culture dishes and left to settle for 75 minutes to allow fibroblasts to attach. The supernatant, containing NRVMs, was then removed and plated onto tissue culture dishes (Ibidi, GmbH) at a density of $4x10^5$ cells/ml in maintenance media (68% DMEM, 17% M199, 5% FBS, 1% fungizone, 1% penstrep (10,000 units/ml), 10% horse serum and 100µM Bromodeoxyuridine (B5002, Sigma) and maintained in a 5% CO_2 incubator at 37°C.[14]

NRVMs were transiently transfected 2-4 days after isolation with either human BIN1 (variant 8, RC220616, Origene Inc), MTM1 (RG205306, Origene Inc) or Tcap (RG203158, Origene Inc) cloned into pCMV6 fluorescent (mKate2, PS100039; mGFP, PS100048; or mBFP,PS100043 respectively) entry vectors or with pCMV6-mKate2 (Origene Inc) as a negative control. Cells were transfected with a 1:3 ratio of Plasmid DNA (6 µg) to

transfection reagent (Fugene 6; E5912, Promega) in reduced serum media (31985062, OptiMEM; Life Technologies, UK) for 48 hours. NRVMs that had been successfully transfected, thus expressing the fluorescent pCMV6 tag, were imaged using a Nikon A1R$^+$ confocal microscope (excitation, 405, 488 or 561 nm; emission, 425-475, 500-530 or 553-618 nm). Nikon Elements imaging software, Manders overlap function, was used to determine the colocalisation of BIN1 with Tcap and MTM1.

T-tubule imaging and analysis – Atrial myocytes were stained with 4 µM di-4-ANEPPS. Additionally, 300µM Fluo 5N (F14203**,** Molecular Probes, ThermoFisher) staining was used to fill atrial t-tubules and 100µM Oregon Green 488 BAPTA-5N (O6812, Molecular Probes, ThermoFisher) staining used to fill NRVM tubules from the bathing solution. T-tubule imaging was performed on either a Leica SP2 confocal microscope at an x-y resolution of 100 nm and vertical z stacks of 162 nm (excitation, 488 nm; emission >515 nm) or a Nikon A1R$^+$ confocal microscope at 100 nm xyz pixel dimensions (excitation, 488 nm; emission 500-530 nm). Where necessary 2 mM EDTA was added to avoid cell movement during the z-stack.

Following image acquisition, confocal stacks were deconvolved, using either Huygens Professional (Scientific Volume Imaging, Netherlands) or NIS elements (Nikon) software. Deconvolution was achieved using the point spread function (PSF) of the microscope. The PSF was calculated 'theoretically' (NIS elements, Nikon) using known microscopic parameters or 'measured' (Huygens Professional, Scientific Volume Imaging) using 100 nm diameter polystyrene beads (Molecular Probes, ThermoFisher Scientific), as described previously.[5, 6, 8] Average fluorescence intensity was then determined from central sections of each cell imaged and confocal stacks thresholded (Image J, NIH).

T-tubule density in sheep atrial cells was assessed in two ways: 1) the fractional area of the cell occupied by t-tubules, calculated from binary converted images, where tubules were represented as a fraction of cell area (Image J, NIH);[8] 2) the distance at which 50% of voxels within the cell are from cell membrane (t-tubule and surface sarcolemma), known as half distance, was calculated using routines written in IDL (Exelis, UK). Distance maps were then calculated whereby, colour intensity represented the distance of each voxel of the cell was from the nearest membrane (Image J, NIH), as described previously.[5, 6, 8]

For the majority of experiments, it was not possible to blind research staff to the disease state of the sheep. However, experiments assessing t-tubule disorder were completed by a blinded observer who was not involved in in-vivo experiments. To assess t-tubule disorder in sheep atrial cells, t-tubules were firstly categorized visually (blinded observations) into normal, mild, moderate or extreme disorder. T-tubule disorder included longitudinal orientation, branching or tubule pairs i.e. deviation from the normal transverse structures located on the z-line. T-tubule orientation was determined using binary images which were "skeletonized" and the Fiji (Image J, NIH) plug-in "directionality" used to generate frequency plots of t-tubule angles.[8] To quantify t-tubule orientation, the sum of t-tubules corresponding to two cell directions (longitudinal versus transverse) was measured and the ratio between the two calculated.[8] Skeletonised images were also used to obtain t-tubule branch information where the Fiji plug-in "analyse skeleton" calculated the average length and the number of elements a tubule structure was comprised of.

Tubule structure was assessed in NRVMs using the Fiji (Image J, NIH) plug-in "Ridge detection" to firstly define tubules (lines of less than 2um were excluded). The images were then "skeletonized" and Fiji plug-in "analyse skeleton" was used to obtain information on t-tubule structures as described above.

Serial Block Face Scanning Electron Microscopy preparation and analysis –Samples of sheep right atrial appendage were fixed in 2.5% glutaraldehyde and 2% paraformaldehyde in 100mM sodium cacodylate buffer, pH 7.2. After washing in sodium cacodylate buffer, samples were prepared as described previously with small modifications.[21] Briefly, samples were subsequently stained in: 2% osmium tetroxide and 1.5% potassium ferrocyanide; 1% thiocarbohydrazide; 2% osmium tetroxide, 1% uranyl acetate, and Walton's lead nitrate with washing in water after each staining step. After staining, samples were dehydrated in an ethanol ascending series (50%, 70%, 90%, 100%, 100%) followed by further propylene oxide dehydration. Increasing concentrations of TAAB 812 hard resin (25%, 50%, 75%, 100%) mixed with propylene oxide were used for infiltration. Finally, samples were embedded in pure resin and cured at 60°C for 36 hours. Samples were extracted from the plastic blocks, glued onto cryo pins, sputter-coated in a gold-palladium alloy and imaged with a Quanta 250 FEG scanning electron microscope, equipped with the Gatan 3View device for serial images collection, and operated at 3.8kV and ~0.46 Torr. Sections were cut at 50 nm and images collected at a nominal resolution of 6.5 -13 nm/px in Gatan .dm4 format. Stacks of images were visualised in Fiji, or 3dmod (IMOD),[21] while image segmentation and volumetric analysis was performed in 3dmod.

Measurement of triggered intracellular Ca^{2+} - Atrial myocytes were loaded with 4µM Fluo-8 AM (1345980-40-6, AAT Bioquest) for 30 minutes. Cells were voltage-clamped using the perforated patch clamp technique AxoClamp2B (Axon Instruments) and pCLAMP software (Molecular Devices, UK) with amphotericin-B (240 µg/ml) in the pipette solution. Cells were stimulated at 0.5Hz using a voltage protocol ramping from -60 to – 40mV and then stepping to +10 mV. Atrial myocytes were imaged confocally using high-speed xyt confocal imaging (Zeiss 7Live; 488 nm excitation and >515 nm emission, at a rate of 128 lines/5 ms). Following acquisition of the time series, 20 µM wheat germ agglutinin ((WGA), W11261, Molecular Probes, ThermoFisher Scientific) was applied to the cells to visualize the t-tubule and surface membranes and confocal z-stacks were recorded at 210 nm xyz pixel dimensions. Experiments were performed at 37°C.

Changes in $[Ca^{2+}]_i$ were measured by Script written in Matlab (Mathworks, UK). ROIs, encompassing the cell width, were selected on the xyt confocal time series images. Fluorescence intensity over time for each ROI was plotted and a correction algorithm was applied to the time series to correct for baseline drift between transients. The time series were spilt into individual transients of 400 frames each, background fluorescence was subtracted and fluorescence was normalised (F/F_0) for each transient. The 'Dyssynchrony' analysis algorithm was then used to process the individual transients, where for each pixel of the cell selected, 50% peak fluorescence or half rise time (TF50) and dys-synchrony (standard deviation of the TF50 values) of the systolic rise of $[Ca^{2+}]_i$ was calculated. Data from three central Ca^{2+} transients in the time series were averaged and used for analysis. To determine the relationship between tubules and Ca^{2+} rise time, the XY coordinates (ImageJ, NIH) for paired data points, distance maps and TF50, were plotted against each other.

Protein Isolation and Immunoblotting – Following removal of the heart (as described above), sheep right atrial appendage tissue samples were snap frozen and stored in liquid nitrogen until use. Samples were homogenised in RIPA buffer containing protease and phosphates inhibitors (0.1 mg/ml phenylmethanesulphonylfluoride; PMSF, 100 mM sodium orthovanadate, 1 mg/ml aprotonin and 1 mg/ml leupetin). Atrial samples were prepared for SDS-PAGE as described previously.[8, 19] Following separation, samples were transferred onto nitrocellulose membranes (GE Healthcare, UK) and blocked using 5% blotto or Superblock (37515, Thermo Scientific,

UK). Membranes were incubated 1:1000 with primary antibodies for BIN1 (sc23918, Santa Cruz Biotechnology), JPH2 (sc51313, Santa Cruz Biotechnology), MTM1(ab128318, Abcam) and Tcap (ab133646, Abcam). HRP conjugated secondary antibodies (sc2005, sc2020, sc2004, Santa Cruz Biotechnology, 1:20,000,) were used with chemiluminescent. Protein levels were normalized to an internal standard (IC) which was loaded on all blots. Each sample was repeated in triplicate and data averaged. Additionally, visualization of Ponceau-S (A40000279, Thermo Fisher Scientific) stained membranes ensured even gel loading and transfer.

Immunocytochemistry– Freshly isolated sheep myocytes from the left atrial appendage were plated onto pre-coated µ-slides or laminin coated cell culture slides (Ibidi, GmbH or Fisher Scientific) and left to settle for a minimum of one hour. Cells were fixed in 4% PFA or 100% acetone for 7-10 minutes prior to incubation with WGA Alexa Fluor® 647 conjugate (1:50, W32466, Thermo Fisher Scientific) for 2 hours at 4°C. Following incubation with WGA, PFA fixed cells were permeabilized with 0.25% Triton x-100 (Sigma) for 10 minutes. Samples were blocked with 10% goat serum followed by incubation with antibodies for NCX (R3F1, Swant, Switzerland) and RyR (ab2827, Abcam) (1:100, diluted in 1% serum) overnight at 4°C. Cells were then stained with goat anti-mouse secondary antibodies (A11001, Molecular Probes, 1:500, diluted in 1% serum) conjugated to Alexa Fluor® 488. Following secondary incubation, slides were imaged using a LeicaSP2 confocal microscope (excitation, 488 or 647 nm; emission 500-530 or 663-738 nm). To validate the specificity of the primary antibodies, secondary antibody-only controls were employed to account for non-specific binding of the secondary antibody. Immunocytochemistry images were digitally deconvolved and thresholded, as described above. Colocalisation analysis was performed (Huygens Professional) to assess the fraction of overlap between NCX, RyR and t-tubules (WGA-Alexa Fluor®) using Manders overlap coefficients.

Statistics – Following enrolment no animals were excluded from the study, n= cells from N = animals / litters. Normality was tested using the Shapiro-Wilk test and normally distributed data is presented as mean ± standard error of the mean (SEM). Where data was not normally distributed, data was transformed (log10) using a method appropriate to the skew of data, or a non-parametric test was used and data presented as median +/- IQR. Where appropriate, data have been compared using linear mixed model analysis, as detailed in the figure legends. A linear mixed model was employed using SPSS Statistics (IBM, USA) with a Restricted Maximum Likelihood (REML) approach. This model included fixed effects for disease (control, HF, recovery) or treatment (transfection of NRVMs) as appropriate, and random effects for animals or isolations (in the case of cell lines). The REML approach was used for estimating the parameters of mixed models (fixed and random effects), as it accounts for the loss of degrees of freedom when estimating fixed effects, resulting in more accurate estimates. Mixed models are useful when data are grouped or clustered, such as repeated measures on the same subjects or measurements from subjects within the same groups. Where data was not normally distributed or when N is small (N<10) normality was supported by larger sample sizes in the literature or differences were assessed using Kruskal-Wallis, Mann-Whitney or Wilcoxon signed-rank tests with multiple comparisons using GraphPad Prism. GraphPad Prism was used to test for data correlations. Exact p values are presented, and data were considered significant when $p<0.05$.

Results

	Pre-pacing	Heart failure	P value (HF vs. control)	Recovery	P value (Rec vs. HF)
Days paced / recovered	-	39.4 ± 3.3	-	37.3 ± 1.2	-
End Diastolic Internal Dimension (cm)	2.88 ± 0.15	4.18 ± 0.22	0.007	3.56 ± 0.20	0.038
End Systolic Internal Dimension (cm)	1.24 ± 0.11	3.32 ± 0.25	0.0003	2.11 ± 0.19	0.005
Fractional area change	0.69 ± 0.01	0.30 ± 0.04	1.5 x10^{-5}	0.58 ± 0.03	1.3 x10^{-5}
Fractional shortening (long axis view)	0.58 ± 0.03	0.21 ± 0.03	1.6 x10^{-5}	0.39 ± 0.03	7.3 x10^{-5}
Cell width (µm)	16.3 ± 0.64	18.62 ± 0.20	0.0009	15.8 ± 1.07	0.030
Cell capacitance (pF)	84.7 ± 3.26	132.5 ± 8.95	0.0001	118.9 ± 9.16	0.35

Supplementary Data, Table 1. Echocardiographic parameters in sheep following tachypacing induced heart failure and cessation of pacing. Cardiac function was improved following cessation of rapid pacing in sheep. Mean data summarizing echocardiographic parameters in sheep. Short-axis and long-axis echocardiogram images were used to calculate left ventricular fractional area change; fractional shortening, end diastolic internal diameter (EDID) and end systolic internal diameter (ESID). Fractional shortening = (EDID – ESID) / EDID. Parasternal images from at least three cardiac cycles were averaged for each measurement. Echocardiographic measurements were taken at each time point for N = 12 animals (9 paired). For cell width measurements; control: n= 31 cells (7 animals), HF: n= 18 (3 animals), recovery: n= 73 cells (7 animals). For cell capacitance measurements; control: n= 53 cells (20 animals), HF n= 41 (16 animals), recovery: n= 25 cells (6 animals) compared using One-way ANOVA with repeated measures.

T-tubule morphology	Description
Column	Uniform in width and long in length.
Angled	Uniform in width and long in length but bent at top (90° ± 45).
Stump	Short in length.
Club	Narrow at base, at least twice as wide at head.
Intermediate	Mid way between stump and fully developed t-tubule.
Cactus	Column with 1 branch - branch is shorter than and usually at an angle to main trunk.
Branched	More than 1 branch and the branch can go in any direction.
Pair	Either the t-tubule splits to run either side of z line or t-tubule is associated with another structure on same z line.
Random	Other – when t-tubule does not fit into the other categories.
Oak tree	Extremely branched with longitudinal elements.
Longitudinal	Runs across several sarcomeres with no main transverse trunk.
Lattice	Grid like pattern.

Supplementary Data, Table 2. Atrial t-tubules were characterized based on morphology. The detailed three-dimensional structure of atrial t-tubules was determined using serial block face Scanning Electron Microscopy (sbfSEM). Reconstructed tubule morphology was defined according to the criteria in Table 2. Both control and recovered atrial t-tubules adopted distinct morphologies which were categorized into 12 groups by an observer.

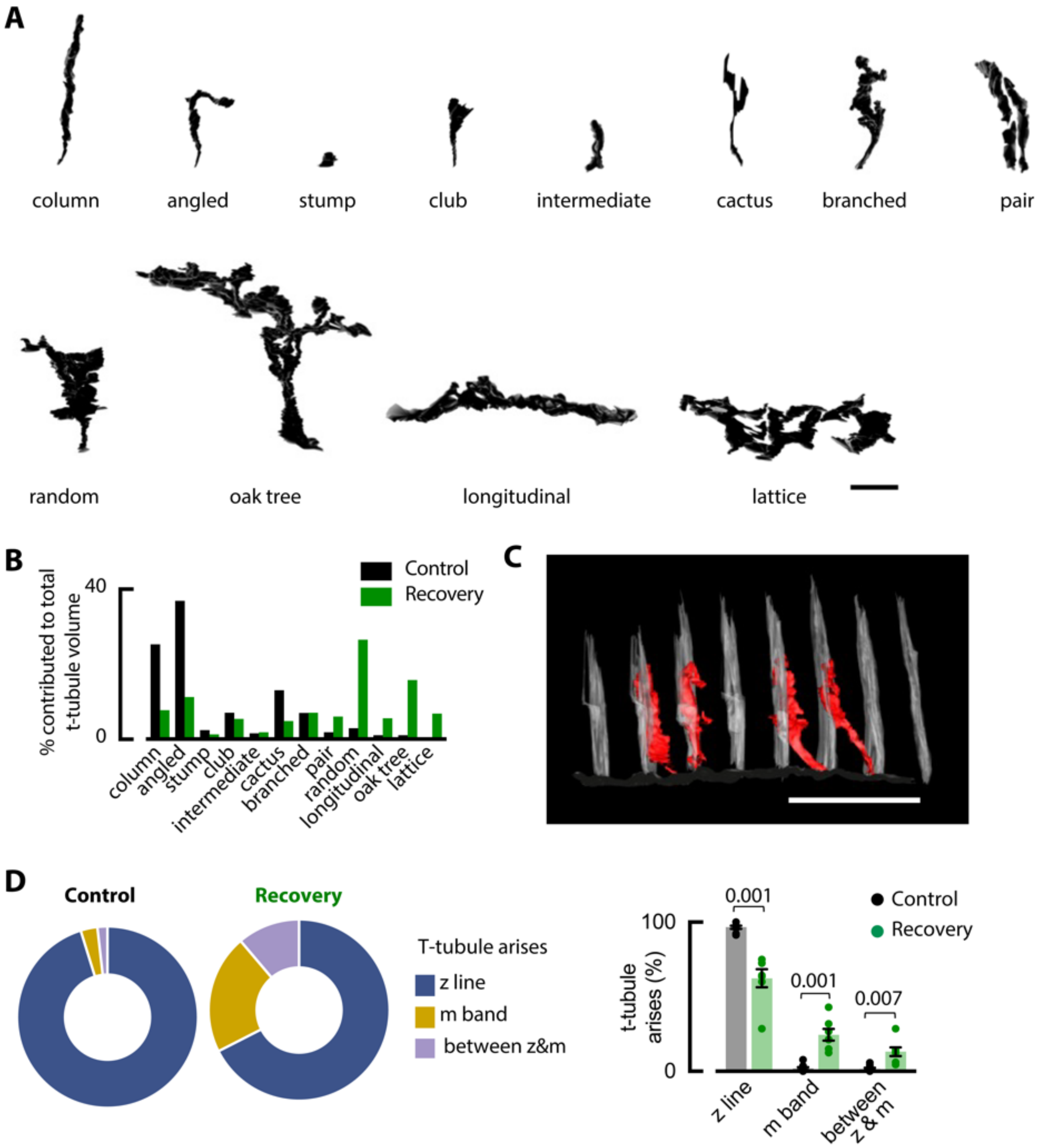

Supplementary Data, Figure 1. T-tubule ultrastructure following recovery from heart failure. (A) 3D reconstructed representative images of the different t-tubule morphologies described in Table 2, scale bar denotes 2µm. (B) Summary data showing % contribution to the total t-tubule volume of each t-tubule morphology in control (black) and recovered (green) atrial myocytes. (C) Atrial t-tubules can span sarcomeres; 3D reconstruction from a sbfSEM image, z-lines are shown in grey and t-tubules shown in red, scale bar denotes 5µm (D) T-tubules arise at non z-line sites more often following recovery from HF. Symbols denote cells; *n* = 9 cells from 3 animals for control and recovery; compared using 2way ANOVA. Data presented as mean +/- SEM.

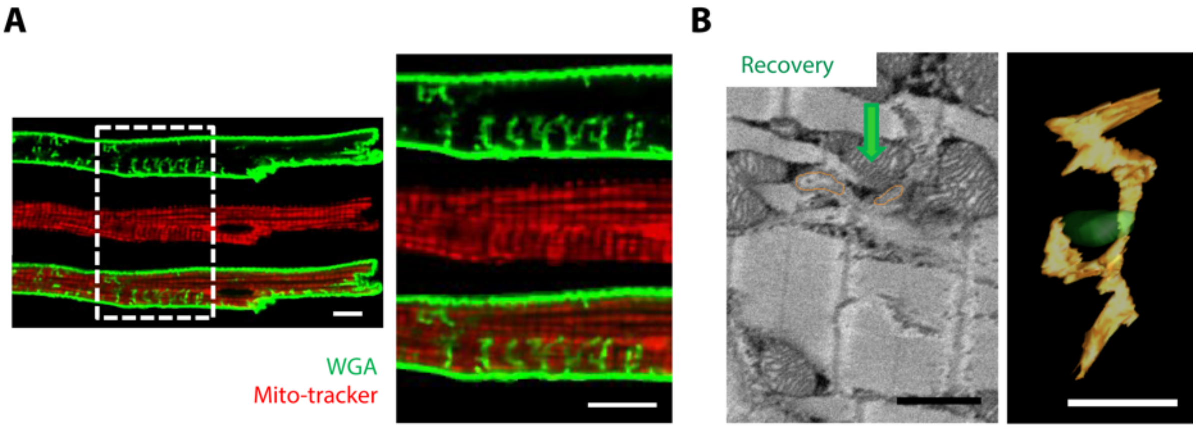

Supplementary Data, Figure 2. Disorder of atrial t-tubules is associated with disrupted mitochondrial positioning. (A) Recovered atrial myocyte imaged confocally showing disordered t-tubules (green) weaving between mitochondria (red); scale bars = 10µm; n = 9 cells from 3 animals for control and recovery. (B) Example 2D-section (left) and model (right) showing a recovered atrial t-tubule (orange) branching around a mitochondrion situated on the z-line (green arrow), scale bars = 2µm.

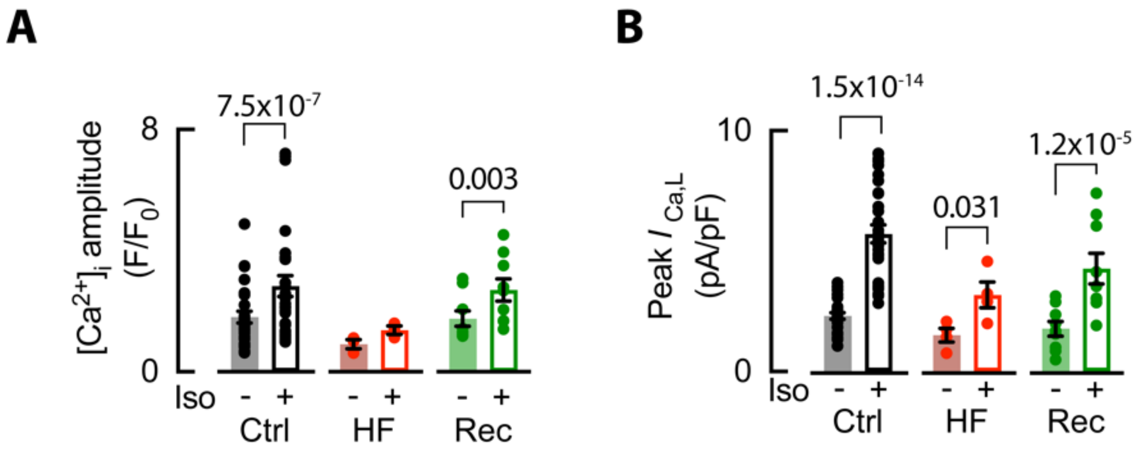

Supplementary Data, Figure 3. Recovered atrial myocytes respond to β-adrenergic stimulation. (A) Isoproterenol (100 nmol/l) increased Ca^{2+} transient amplitude in control and recovery atrial myocytes. (B) Peak $I_{Ca\text{-}L}$ following application of isoproterenol (100 nmol/l) in control, HF and recovery atrial myocytes. Symbols denote cells *n;* Control: *n*= 27 (7 animals); HF: *n*= 3 for panel A, *n*= 4 for panel B (3 animals); Recovery: *n*= 9 (5 animals) (control and HF to confirm our previous work[19]); compared using 2way RM ANOVA. Data presented as mean +/- SEM.

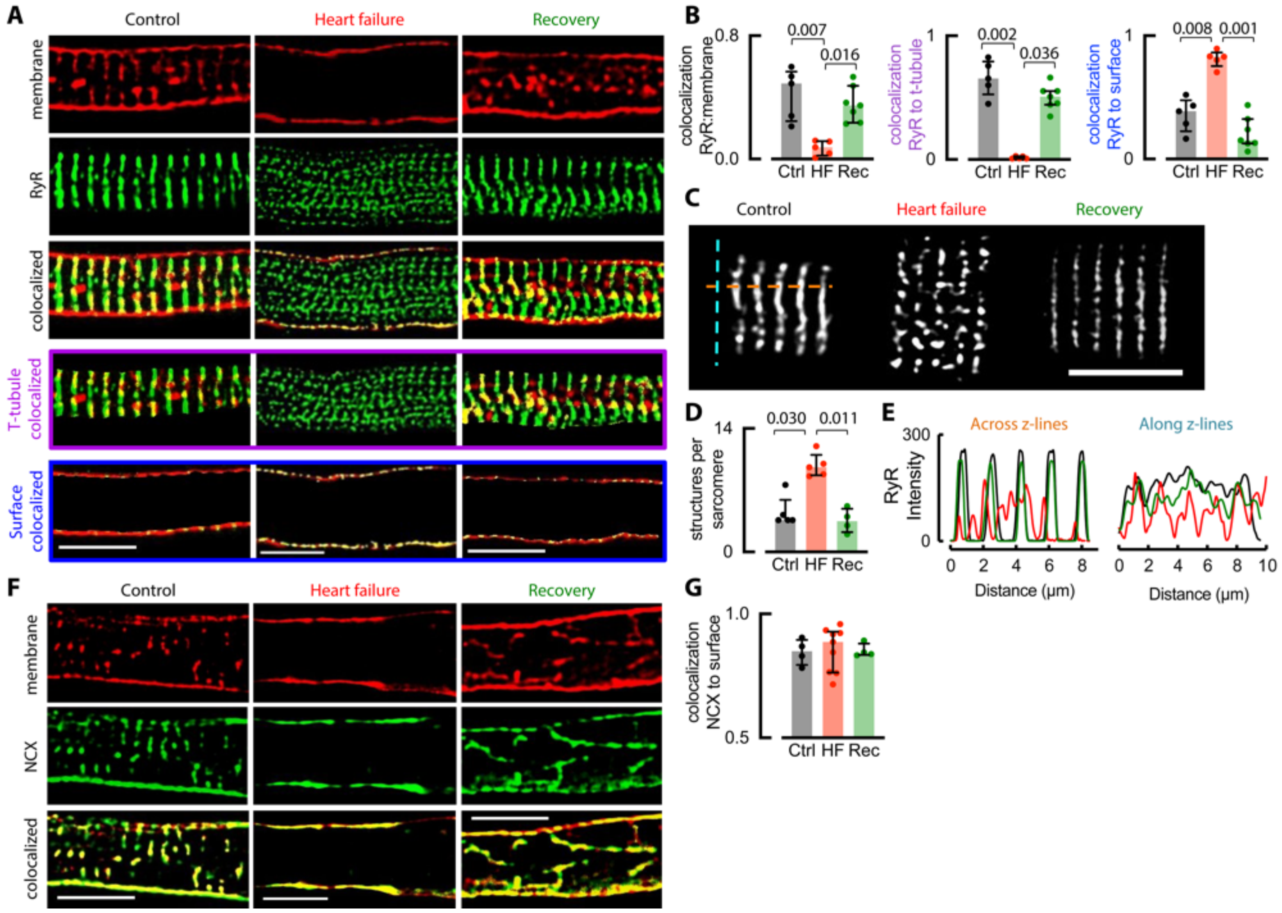

Supplementary Data, Figure 4. Cellular distribution of Ca handling proteins. (A) Immuno staining of RyR (green) and tubules or surface membrane (membrane - red), yellow represents colocalisation (B) Mean data showing Manders colocalisation of; RyR and t-tubules with or without surface membrane; surface RyR (independent of cytosolic RyR) and surface membrane in control (Ctrl), HF and recovery atrial myocytes. (C) RyR distribution along the sarcomere. (D) Mean data for number of RyR structures per sarcomere. (E) representative intensity blots across RyR stained sarcomeres from (C) in both longitudinal and transverse directions. (F) Immuno staining of NCX (green) and tubules or surface membrane (membrane - red) in atrial myocytes, yellow represents colocalisation. (G) Mean data showing Manders colocalisation of; NCX and t-tubules (membrane). Symbols denote cells *n*; NCX, n= 4 control, n= 9 HF, n= 4 recovery cells; for RyR n= 5 control, n= 5 HF, n= 7 recovery cells from 2 animals per group; compared using Kruskai-Wallis test with multiple comparisons. Data presented as median +/- IQR, scale bars = 10μm.

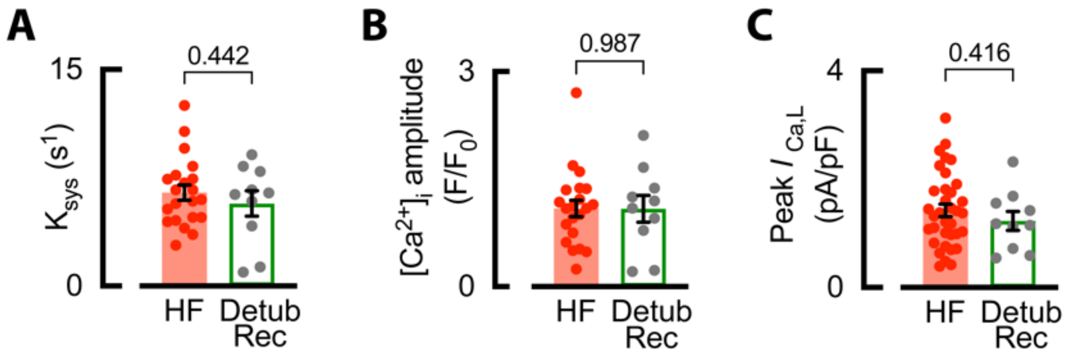

Supplementary Data, Figure 5. The removal of recovered t-tubules reverted the mean rate of decay of the systolic Ca^{2+} transient (k_{SYS}); mean systolic Ca^{2+} transient amplitude and mean peak $I_{Ca\text{-}L}$ to HF levels. Symbols denote cells *n*, HF: *n*= 21 (9 animals) for panels A-B, *n*= 35 (14 animals) for panel C; Detubulated: *n*= 10 (4 animals); compared using linear mixed modelling. Data taken from Figure 4, 6, 7. Data presented as mean +/- SEM.

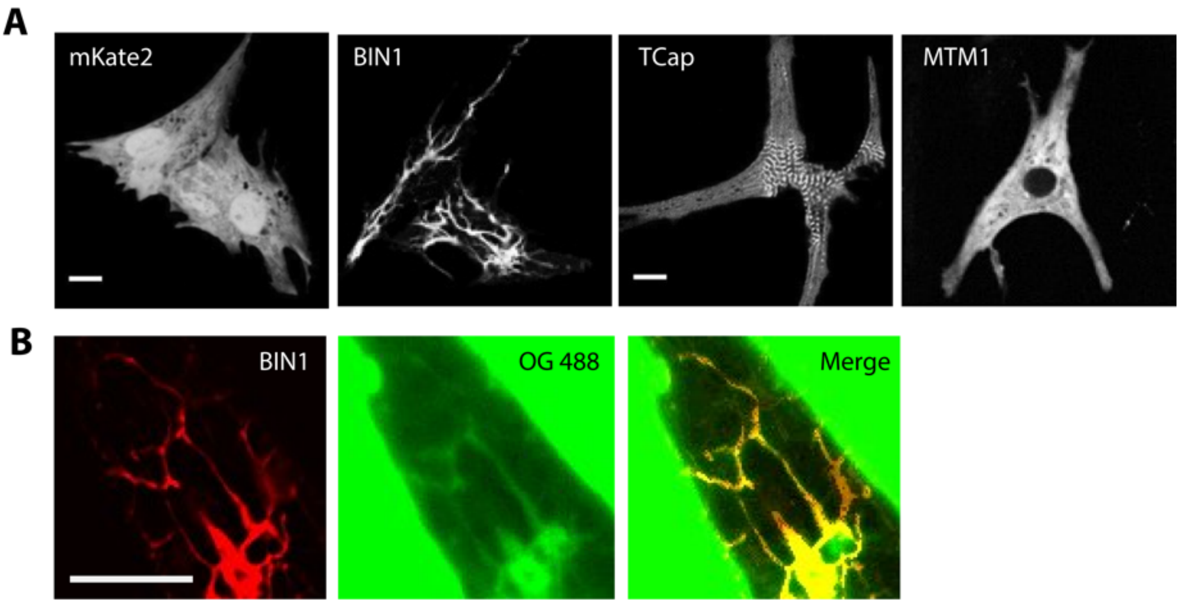

Supplementary Data, Figure 6. Expression of proteins likely to increase t-tubule abundance. (A) Representative NRVMs transfected with mKate2 (empty vector), BIN1, MTM1 or Tcap expression vectors. (B) Oregon Green (green) staining of BIN1 transfected cell (red) and merge (yellow). Scale bars = 10µm.
