# Supplementary material for: Restoring Atrial T-Tubules Augments Systolic Ca Upon Recovery From Heart Failure
Source: Circ Res. 2024 Aug 14;135(7):739–54. doi: 10.1161/CIRCRESAHA.124.324601 (PMC11392124; doi:10.1161/CIRCRESAHA.124.324601)
Supplement: Supplementary file 3 [file res-135-739-s003.pdf]

Major Resources Table

In order to allow validation and replication of experiments, all essential research materials listed in the Methods should be included in the Major Resources Table below. Authors are encouraged to use public repositories for protocols, data, code, and other materials and provide persistent identifiers and/or links to repositories when available. Authors may add or delete rows as needed.

Animals (in vivo studies)

Species	Vendor or Source	Background Strain	Sex	Persistent ID / URL
Sheep	Biological Services Facility	Welsh mountain	Female	
Rat	Charles River UK ltd	Wistar rats	Female (pregnant)	https://www.criver.com/products-services/find-model/wistar-igs-rat?region=3611

Antibodies

Target antigen	Vendor or Source	Catalog #	Working concentration	Persistent ID / URL
BIN1	Santa Cruz Biotechnology	sc23918	1:1000	https://www.scbt.com/p/amphiphysin-ii-antibody-2f11
JPH2	Santa Cruz Biotechnology	sc51313	1:1000	https://www.scbt.com/p/junctophilin-2-antibody-y-15
MTM1	Abcam	ab128318	1:1000	https://www.abcam.com/products/primary-antibodies/mtm1-antibody-ab128318.html
Tcap	Abcam	ab133646	1:1000	https://www.abcam.com/products/primary-antibodies/telethonin-antibody-epr8375-ab133646.html
NCX	Swant, Switzerland	R3F1	1:100	https://webshop.swant.com/r3f1-na-ca2-exchanger-protein-cardiac.html
RyR	Abcam	ab2827	1:100	https://www.abcam.com/products/primary-antibodies/ryanodine-receptor-antibody-c3-33-ab2827.html
secondary antibody for BIN1	Santa Cruz Biotechnology	sc2005	1:20000	https://www.scbt.com/p/goat-anti-mouse-igg-hrp
secondary antibody for JPH2	Santa Cruz Biotechnology	sc2020	1:20000	https://www.scbt.com/p/donkey-anti-goat-igg-hrp
secondary antibody for MTM1 and Tcap	Santa Cruz Biotechnology	sc2004	1:20000	https://www.scbt.com/p/goat-anti-rabbit-igg-hrp
secondary antibody for NCX and RyR	Molecular Probes, ThermoFisher	A11001	1:500	https://www.thermofisher.com/antibody/product/Goat-anti-Mouse-IgG-H-L-Cross-Adsorbed-Secondary-Antibody-Polyclonal/A-11001

Plasmids/Expression Vectors

Vector Name	Catalog #	Vendor or Source	Persistent ID / URL
BIN1 (Myc-DDK-tagged)-Human bridging integrator 1 (BIN1), transcript variant 8	RC220616	Origene	https://www.origene.com/catalog/cdna-clones/expression-plasmids/rc220616/bin1-nm_004305-human-tagged-orf-clone

DOI [to be added]

TCAP (tGFP-tagged) - Human titin-cap (telethonin) (TCAP)	RG203158	Origene	https://www.origene.com/catalog/cdna-clones/expression-plasmids/rg203158/telethonin-tcap-nm_003673-human-tagged-orf-clone
MTM1 (NM_000252) Human Tagged ORF Clone	RG205306	Origene	https://www.origene.com/catalog/cdna-clones/expression-plasmids/rg205306/mtm1-nm_000252-human-tagged-orf-clone
pCMV6-AC-mKate	PS100039	Origene	https://www.origene.com/catalog/vectors/mammalian-expression-vectors/ps100039/pcmv6-ac-mkate-mammalian-expression-vector
pCMV6-AC-mBFP	PS100043	Origene	https://www.origene.com/catalog/vectors/mammalian-expression-vectors/ps100043/pcmv6-ac-mbfp-mammalian-expression-vector
pCMV6-AN-mGFP	PS100048	Origene	https://www.origene.com/catalog/vectors/mammalian-expression-vectors/ps100048/pcmv6-an-mgfp-mammalian-expression-vector
**Vectors generated at UOM**	**Cloned into**	**Insert**	**Sequence**
pCMV6-AC-mKate- BIN1 v8	PS100039	BIN1 From RC220616	See Origene RC220616
pCMV6-AC-mBFP-Tcap	PS100043	Tcap from RG203158	See Origene RG203158
pCMV6-AN-mGFP–MTM1	PS100048	MTM1 from RG205306	See Origene RG205306

Cultured Cells

Name	Vendor or Source	Sex (F, M, or unknown)	Persistent ID / URL
Neonatal rat ventricular myocytes	Isolated in house from two-day old Wistar rats (Charles River UK ltd)	Both	n/a

Data & Code Availability

Description	Source / Repository	Persistent ID / URL

Other

Description	Source / Repository	Persistent ID / URL

DOI [to be added]
