# Supplementary material for: Restoring Atrial T-Tubules Augments Systolic Ca Upon Recovery From Heart Failure
Source: Circ Res. 2024 Aug 14;135(7):739–54. doi: 10.1161/CIRCRESAHA.124.324601 (PMC11392124; doi:10.1161/CIRCRESAHA.124.324601)
Supplement: Supplementary file 4 [file res-135-739-s004.pdf]

Full unedited gel for Figure 8A (BIN1, top panel)

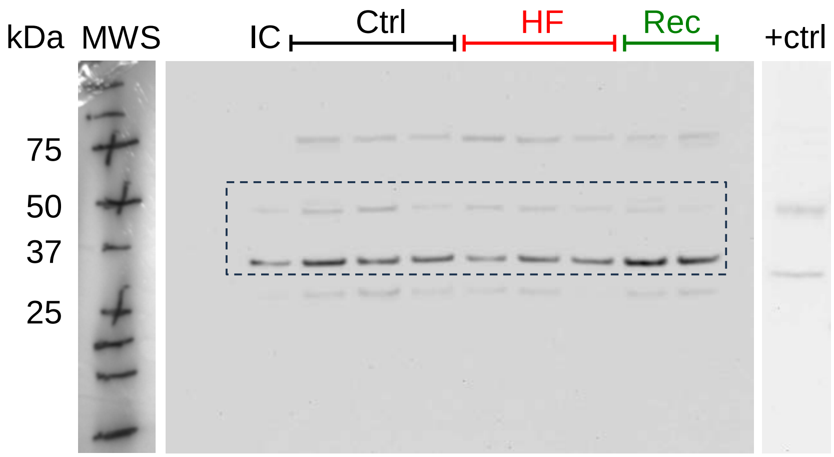

Full unedited gel for Figure 8A (BIN1, top panel). Dashed line corresponds to lanes shown in the cropped images within the manuscript. Positive control (+ctrl) of rat ventricular muscle was used to confirm our previous work,[8] and antibody selectivity. Prestained molecular weight standard (MWS) denotes the location of native molecular weights. Protein levels were normalized to an internal standard (IC) which was loaded on all blots. Each sample was repeated in triplicate and data averaged.

Full unedited gel for Figure 8A (JPH2, second panel)

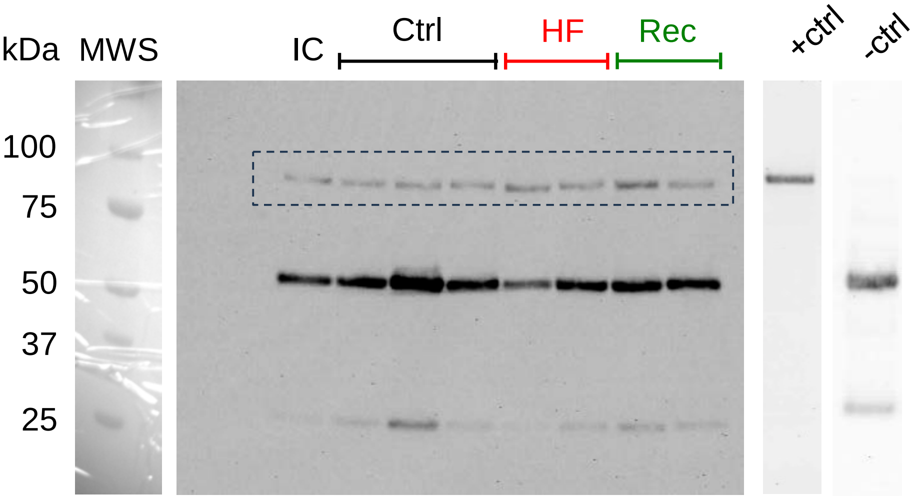

Full unedited gel for Figure 8A (JPH2, second panel). Dashed line corresponds to lanes shown in the cropped images within the manuscript. Positive control (+ctrl) of rat ventricular muscle, as recommended per manufacturer and negative control of secondary antibody only was used to confirm antibody selectivity. Prestained molecular weight standard (MWS) denotes the location of native molecular weights. Protein levels were normalized to an internal standard (IC) which was loaded on all blots. Each sample was repeated in triplicate and data averaged.

Full unedited gel for Figure 8A (Tcap, third panel)

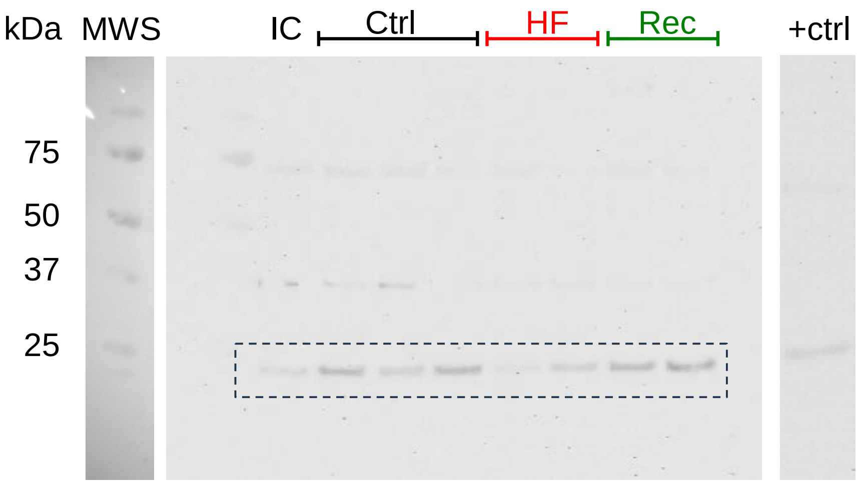

Full unedited gel for Figure 8A (Tcap, third panel). Dashed line corresponds to lanes shown in the cropped images within the manuscript. Positive control of sheep fetal heart lysate, as recommended per manufacturer , was used to confirm antibody selectivity. Prestained molecular weight standard (MWS) denotes the location of native molecular weights. Protein levels were normalized to an internal standard (IC) which was loaded on all blots. Each sample was repeated in triplicate and data averaged.

Full unedited gel for Figure 8A (MTM1, bottom panel)

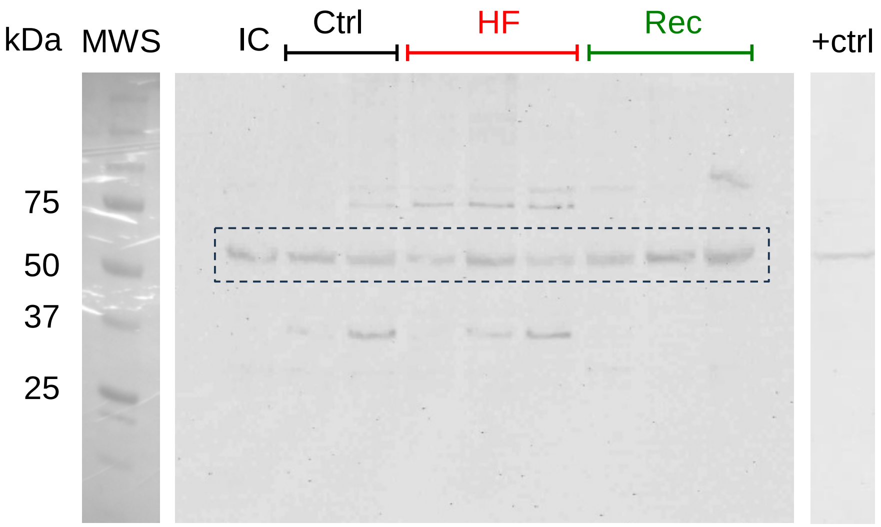

Full unedited gel for Figure 8A (MTM1, bottom panel). Dashed line corresponds to lanes shown in the cropped images within the manuscript. Positive control of mouse liver tissue, as recommended per manufacturer, was used to confirm antibody selectivity. Prestained molecular weight standard (MWS) denotes the location of native molecular weights. Protein levels were normalized to an internal standard (IC) which was loaded on all blots. Each sample was repeated in triplicate and data averaged.
